# Supplementary material for: Adipocyte heterogeneity regulated by the Bithorax Complex-Wnt signaling crosstalk in Drosophila
Source: EMBO Rep. 2025 Dec 3;27(2):367–86. doi: 10.1038/s44319-025-00625-z (PMC12852179; doi:10.1038/s44319-025-00625-z)
Supplement: Supplementary file 1 — Appendix [file 44319_2025_625_MOESM1_ESM.pdf]

Adipocyte heterogeneity regulated by the Bithorax Complex-Wnt signaling crosstalk in *Drosophila*

Rajitha-Udakara-Sampath Hemba-Waduge, Mengmeng Liu, Xiao Li, Jasmine L. Sun, Elisabeth A. Budslick, Sarah E. Bondos, and Jun-Yuan Ji

Table of Contents

Appendix Figure S1 -- page 2
Appendix Figure S2 -- page 3
Appendix Figure S3 -- page 4
Appendix Figure S4 -- page 5
Appendix Figure S5 -- page 6
Appendix Figure S6 -- page 7
Appendix Figure S7 -- page 8
Appendix Figure S8 -- page 9
Appendix Figure S9 -- page 10
Appendix Figure S10 -- page 10
Appendix Figure S11 -- page 11
Appendix Figure S12 -- page 12
Appendix Figure S13 -- page 13
Appendix Figure S14 -- page 14
Appendix Figure S15 -- page 15
Appendix Figure S16 -- page 16
Appendix Figure S17 -- page 17
Appendix Figure S18 -- page 18

Appendix Table S1 -- page 19
Appendix Table S2 -- page 20

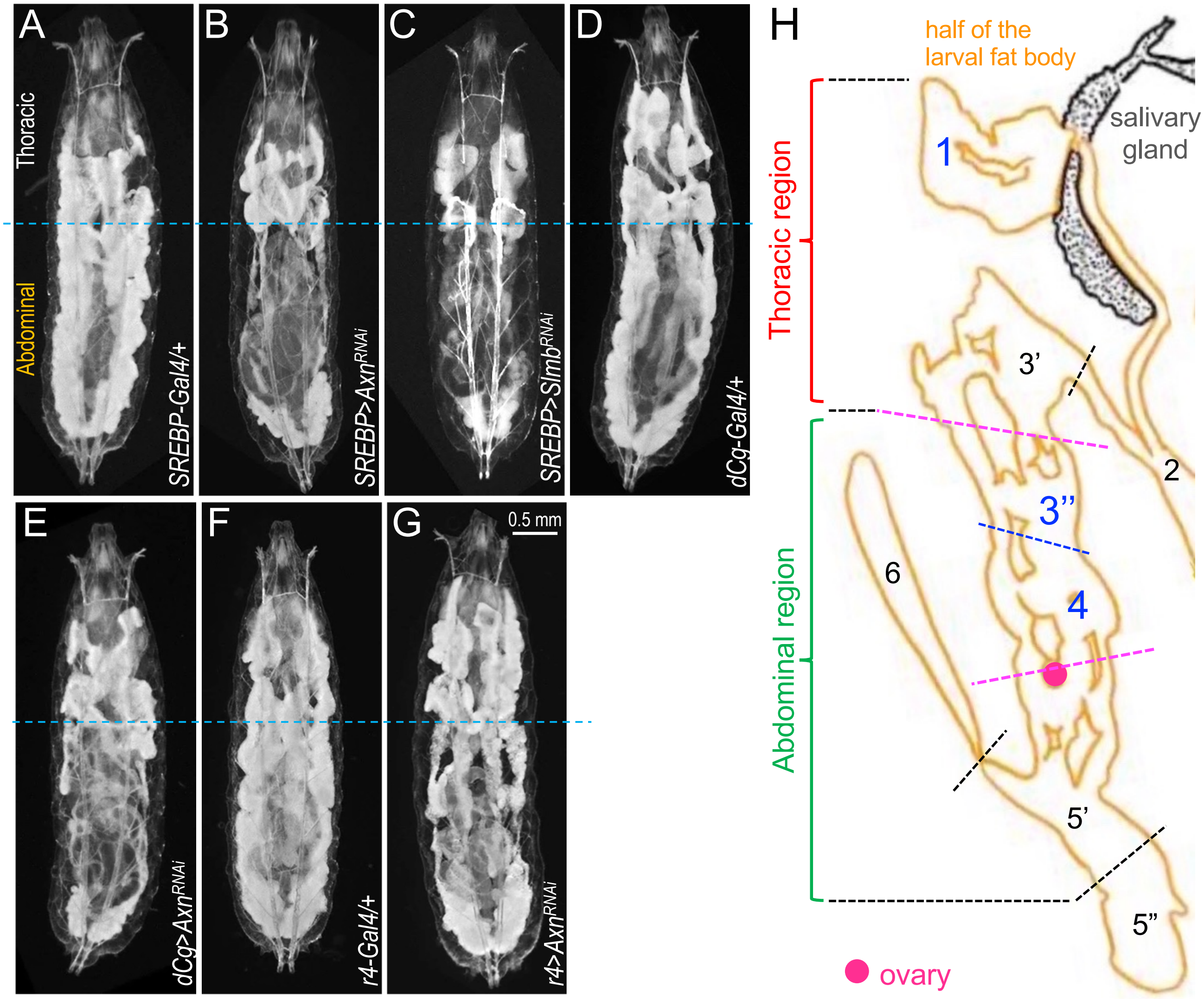

Appendix Figure S1. Whole larvae images showing the depletion of *Axn* or *slmb* using several fat body-specific Gal4 drivers. Note the specific reduction in abdominal fat accumulation. Genotypes are as follows: (A) +*; SREBP-Gal4*/+; (B) *UAS-Axn*^{RNAi}/+*; SREBP-Gal4*/+; (C) +*; SREBP-Gal4/UAS-Slmb*^{RNAi}; (D) *dCg-Gal4*/+*;* +; (E) *dCg-Gal4/UAS-Axn*^{RNAi}*;* +; (F) +*; r4-Gal4*/+; and (G) *UAS-Axn*^{RNAi}/+*; r4-Gal4*/+. Scale bar in panel (G): 0.5 mm. (H) Schematic diagram showing six distinct segments of the larval fat body (adapted from Rizki, T.M. (1964) *J. Cell Biol.* 21, 203-211). Regions 1 to 3' correspond to the thoracic region of the fat body (Region 1 was used for RNA-seq experiments and confocal imaging). Regions 3'' to 5' and 6 correspond to the abdominal region of the fat body (Regions 3''-5' and 6 were used for RNA-seq experiments, while Region 4 was used for confocal imaging). The strawberry-colored dot indicates the position of the ovary. For clarity, only the left half of the fat body is shown. SG refers to salivary glands.

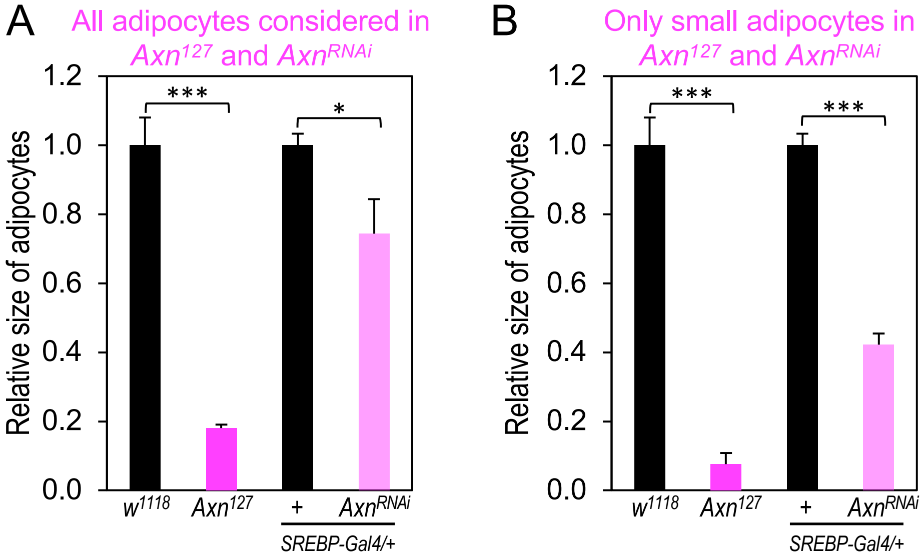

Appendix Figure S2. Adipocyte size quantifications for *Axn*^{127} and *Axn*^{RNAi}. Respective adipocyte size quantifications considering all adipocytes (A) and Wnt-active small adipocytes (B) in *Axn*^{127} and *Axn*^{RNAi} (n = 3, independent biological repeats). $p<0.05$ (*), and $p<0.001$ (***); based on one-tailed unpaired *t*-tests because our experimental model is based on well-defined directional predictions regarding Wnt signaling outcomes.

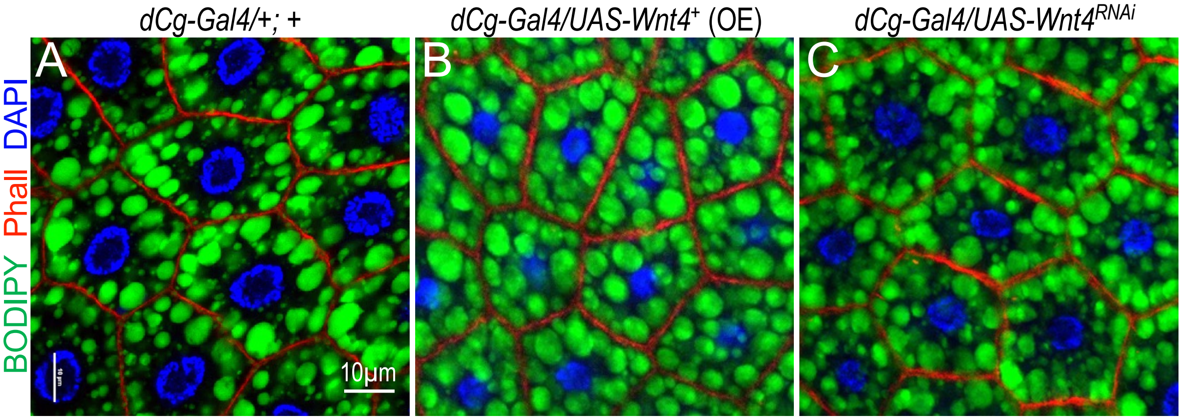

Appendix Figure S3. Ectopic expression or depletion of *Wnt4* does not result in obvious phenotypic effects in adipocytes. (A-C) Representative confocal images of larval adipocytes from larvae with the indicated genotypes, stained with DAPI (blue), BODIPY (green), and Phalloidin (Phall; red). Genotypes are as follows: (A) *dCg-Gal4/+; +*, (B) *dCg-Gal4/UAS-Wnt4^{+}; +* (OE: overexpression), and (C) *dCg-G4/+; UAS-Wnt4^{RNAi}/+*. The scale bar in (A): 10 µm.

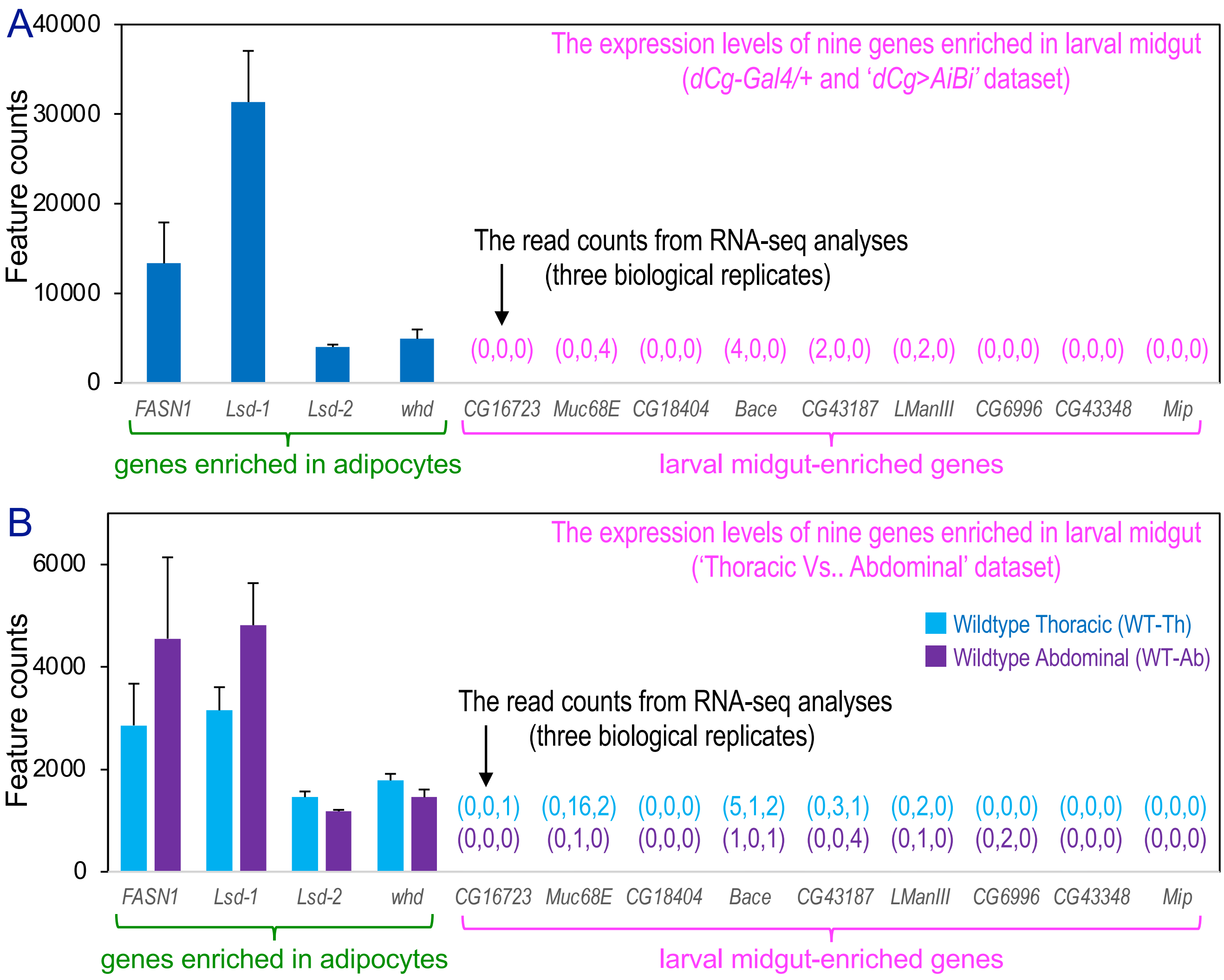

Appendix Figure S4. Bar charts showing feature counts from RNA-seq data for adipocyte-expressed and larval midgut-specific genes. Expression levels are shown for four adipocyte-enriched genes (*FASN1*, *Lsd-1*, *Lsd-2*, *whd*) and nine midgut-enriched genes that were detected in RNA-seq datasets derived from dissected fat bodies. (A) Feature counts from the *dCg-Gal4/+* control sample in the *dCg>AiBi* dataset. (B) Feature counts from wild-type thoracic (WT-Th) and wild-type abdominal (WT-Ab) fat body samples. The read counts from RNA-seq analyses (three biological replicates) are shown above each of the nine genes enriched in the larval intestine.

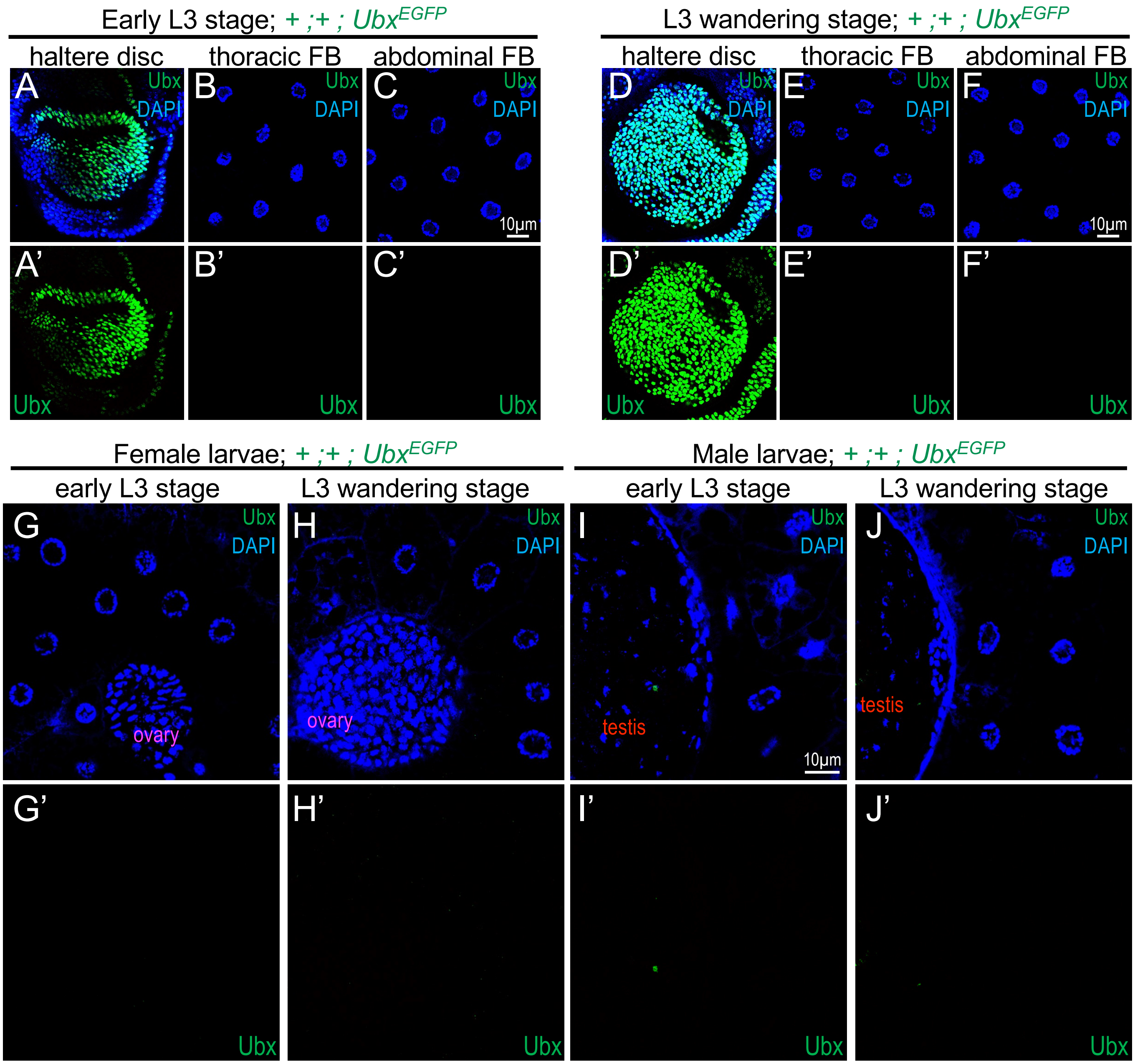

Appendix Figure S5. Endogenous expression pattern of EGFP-tagged Ubx (Ubx^{EGFP}). The endogenous expression pattern of Ubx^{EGFP} in haltere discs (A/A', D/D'), the thoracic region of the larval FB (fat body) (B/B' and E/E'), and the abdominal region of the fat body (C/C' and F/F') from early L3 stage (A-C) and L3 wandering stage (D-F) larvae. The endogenous expression pattern of Ubx^{EGFP} in female ovaries (G/G' and H/H') and male testes (I/I' and J/J') from early L3 stage (G/G', I/I') and L3 wandering stage (H/H', J/J') larvae. Ubx^{EGFP} is shown in green, and DAPI (blue) stains the nuclei. The scale bars in panel (C/F/I): 10 μm.

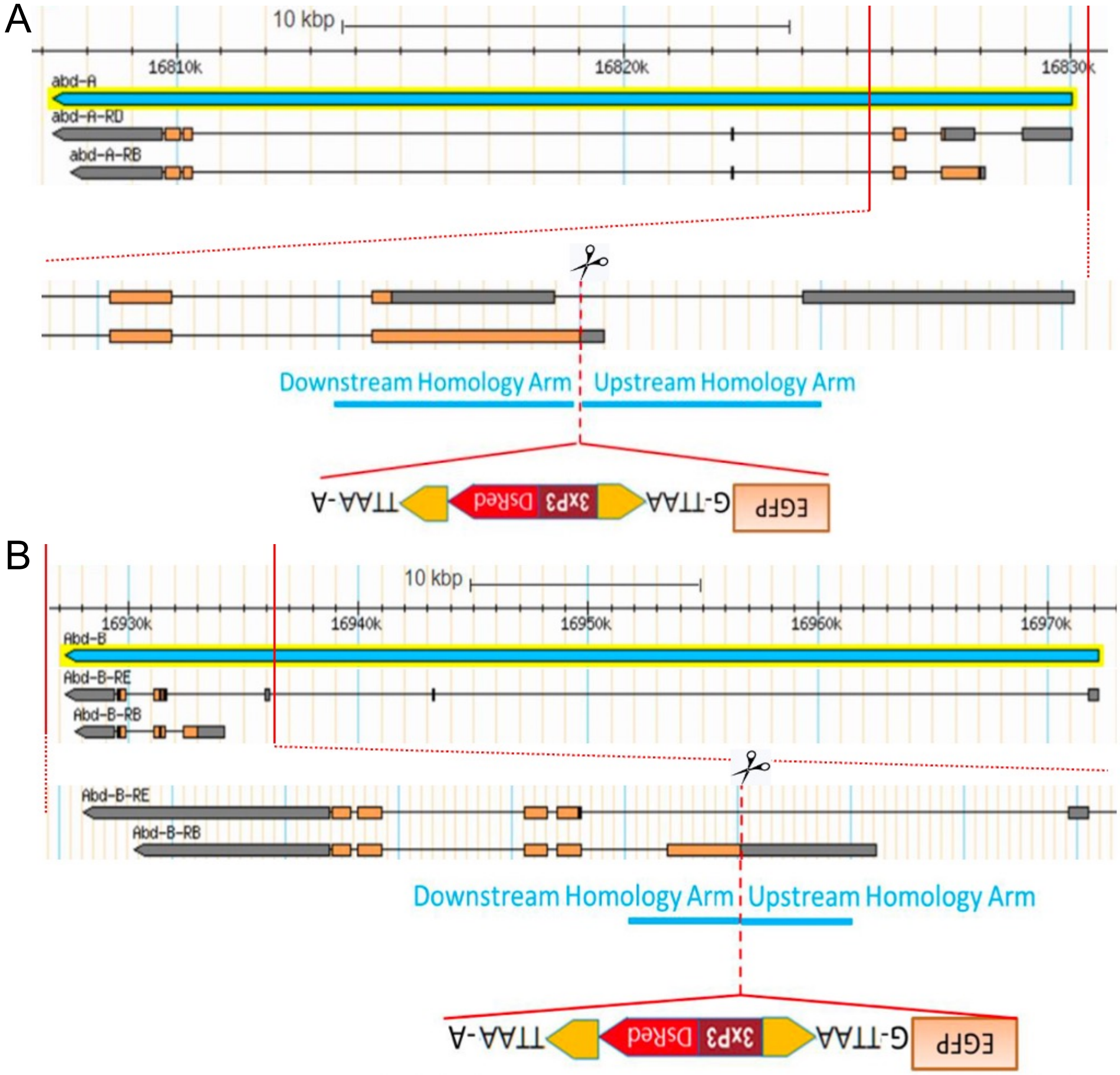

Appendix Figure S6. CRISPR-Cas9 based EGFP-tagging of *abd-A* and *Abd-B*. CRISPR/Cas9-mediated genome editing via homology-dependent repair (HDR) was performed using one guide RNA and a dsDNA plasmid donor. The N-termini of Abd-A (RB form) (A) and Abd-B (RB form) (B) were tagged using the “EGFP-PBacDsRed” cassette, which contains EGFP and 3xP3-DsRed flanked by PiggyBac terminal repeats. Two homology arms were cloned into pUC57-Kan as donor templates for repair. Scale bar: 10 kb.

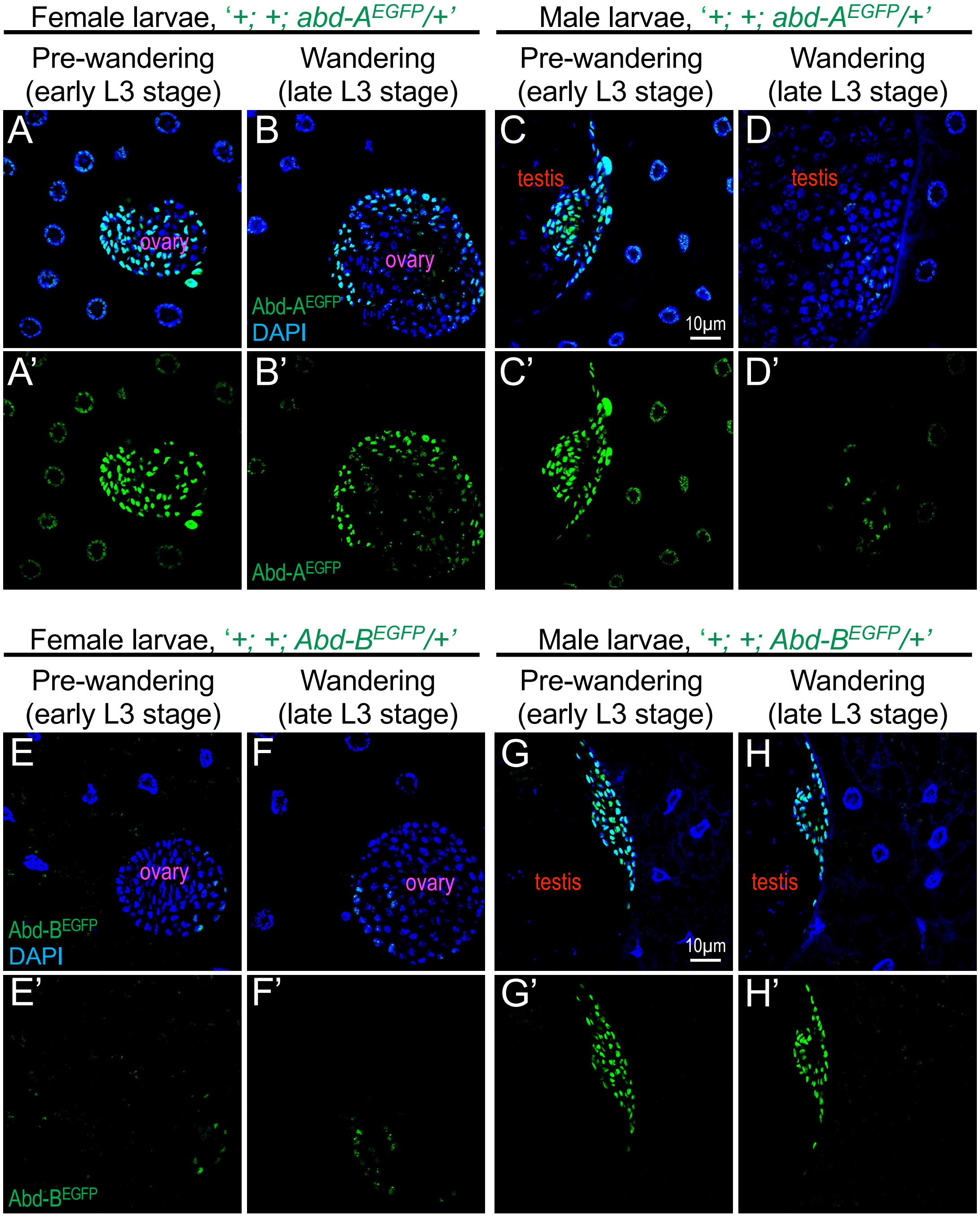

Appendix Figure S7. Endogenous expression pattern of EGFP-tagged Abd-A (Abd-A^{EGFP}) and Abd-B (Abd-B^{EGFP}). The endogenous expression pattern of Abd-A^{EGFP} in ovaries (A/A', B/B') and testes (C/C', D/D') from early L3 stage (A/A', C/C') and late L3 stage (B/B', D/D') larvae. The endogenous expression pattern of Abd-B^{EGFP} in ovaries (E/E', F/F') and testes (G/G', H/H') from early L3 stage (E/E', G/G') and late L3 stage (F/F', H/H') larvae. Abd-A^{EGFP}/Abd-B^{EGFP} are shown in green, and DAPI (blue) stains the nuclei. The scale bars in panel (C/G): 10 μm.

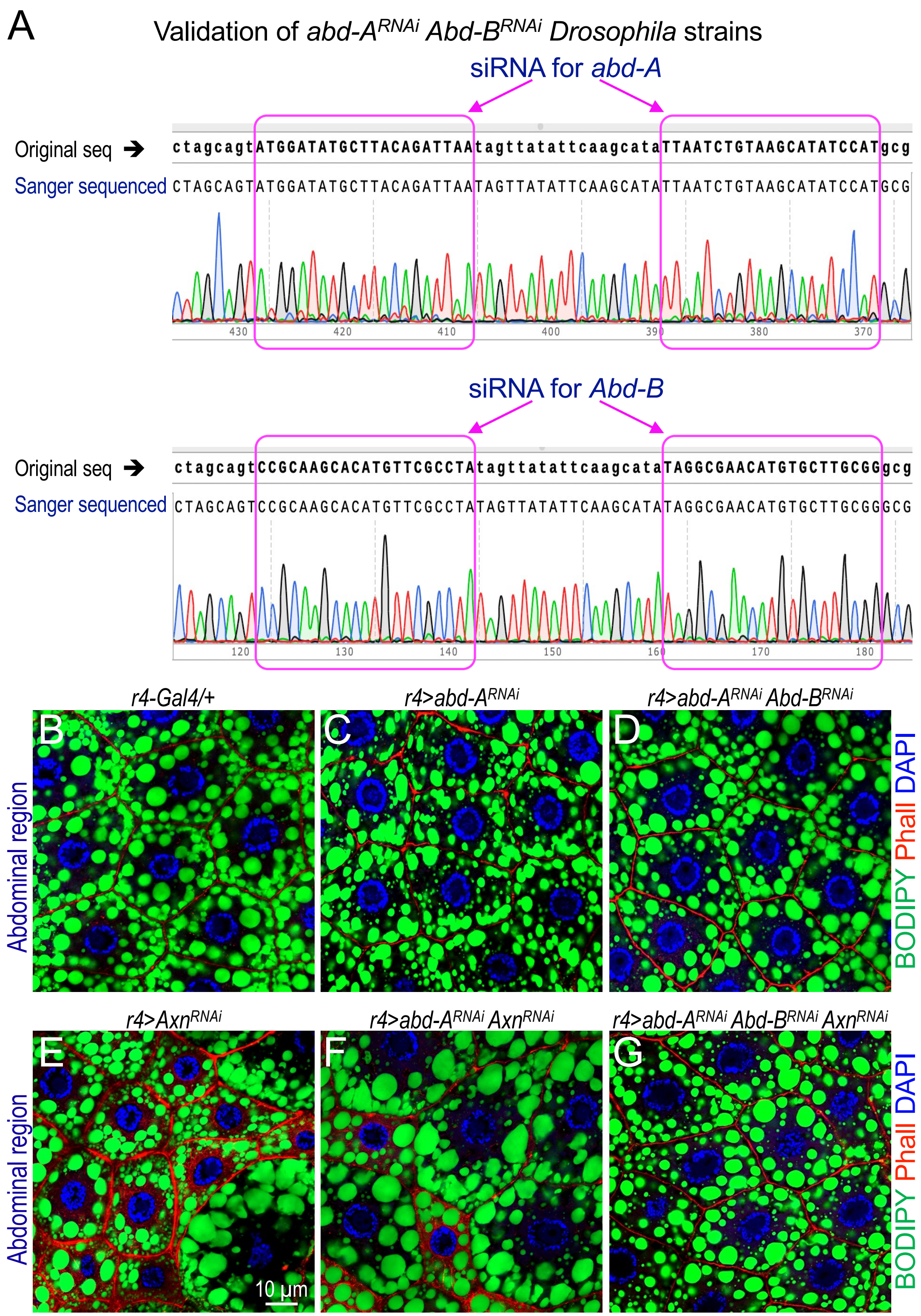

Appendix Figure S8. Validation of *abd-A*^{RNAi} *Abd-B*^{RNAi} *Drosophila* strains. (A) Sequence validation of pNP-based *abd-A* and *Abd-B* double depletion lines using Sanger sequencing. (B-G) Depleting *abd-A* and/or *Abd-B* under active Wnt signaling background using pNP vector-based system. Genotypes are as follows: (B) +*; r4-Gal4*/+; (C) $_{pNP}$*UAS-Abd-A*^{RNAi}/+*; r4-Gal4*/+; (D) $_{pNP}$*UAS-Abd-A*^{RNAi} *Abd-B*^{RNAi}/+*; r4-Gal4*/+; (E) *UAS-Axn*^{RNAi}/+*; r4-Gal4*/+; (F) $_{pNP}$*UAS-Abd-A*^{RNAi}/*UAS-Axn*^{RNAi}*; r4-Gal4*/+; and (G) $_{pNP}$*UAS-Abd-A*^{RNAi} *Abd-B*^{RNAi}/*UAS-Axn*^{RNAi}*; r4-Gal4*/+. The scale bar in panel (E): 10 μm.

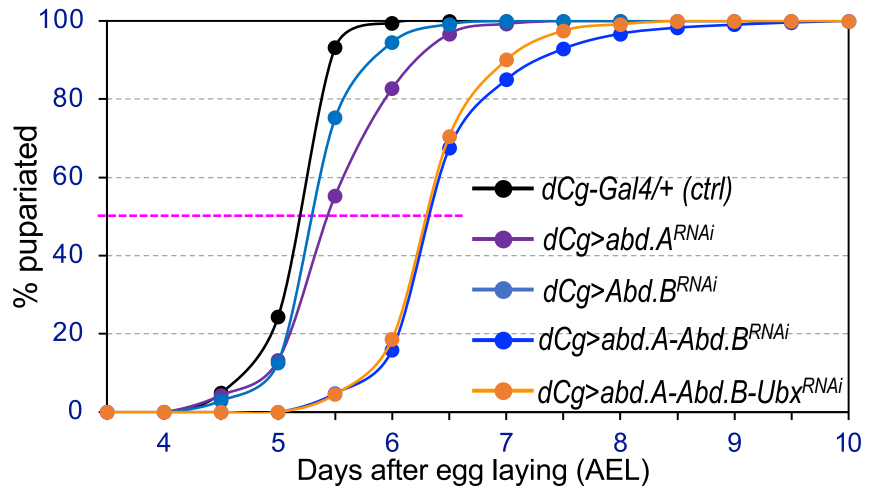

Appendix Figure S9. Effects of Abd-A and Abd-B depletion on larval pupariation and lipid accumulation in adipocytes. Pupariation curves showing the percentage of larvae that pupariated over time, measured in days after egg laying (AEL). Genotypes are as follows: *dCg-Gal4/+; +* (black), *dCg-Gal4/+; UAS-abd-A*^{RNAi}/+ (purple), *dCg-Gal4/+; UAS-abd-B*^{RNAi}/+ (pale blue), *dCg-Gal4/pNP-[abd-A Abd-B]*^{RNAi}*;+* (dark blue), *dCg-Gal4/pNP-[abd-A Abd-B Ubx]*^{RNAi}*;+* (orange).

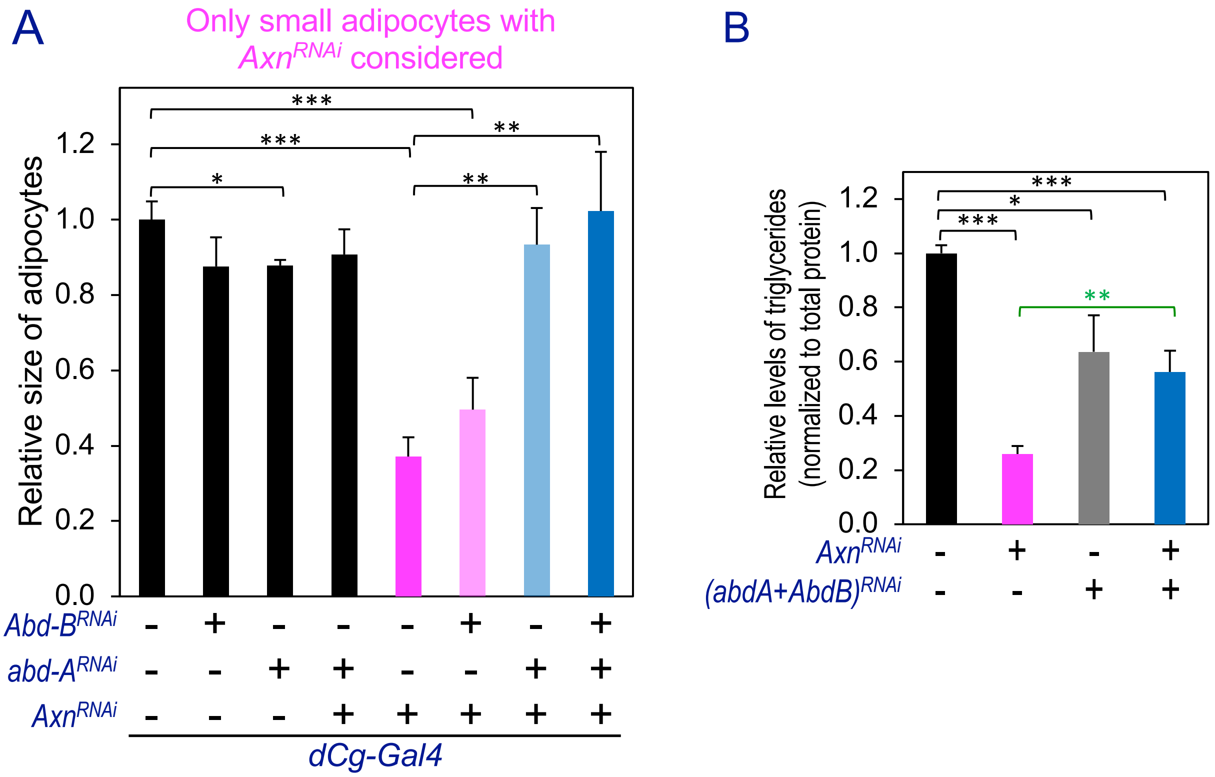

Appendix Figure S10. Systematic quantification of adipocyte sizes and triglyceride levels upon depleting *abd-A* and *Abd-B* under Wnt-activated background. Adipocyte quantification considering Wnt-active small adipocytes (A) in Axn^{RNAi}. Different genetic combinations of *abd-A*, *Abd-B* and *Axn* depletions are shown below. (B) Relative triglyceride levels in larvae with *abd-A/Abd-B* and/or *Axn* depletion in the larval fat body. Triglyceride (TG) levels were measured using a commercial TG quantification kit (n = 3, independent biological repeats). p<0.05 (*), p<0.01 (**), and p<0.001 (***); based on one-tailed unpaired *t*-tests because our experimental model is based on well-defined directional predictions regarding Wnt signaling outcomes.

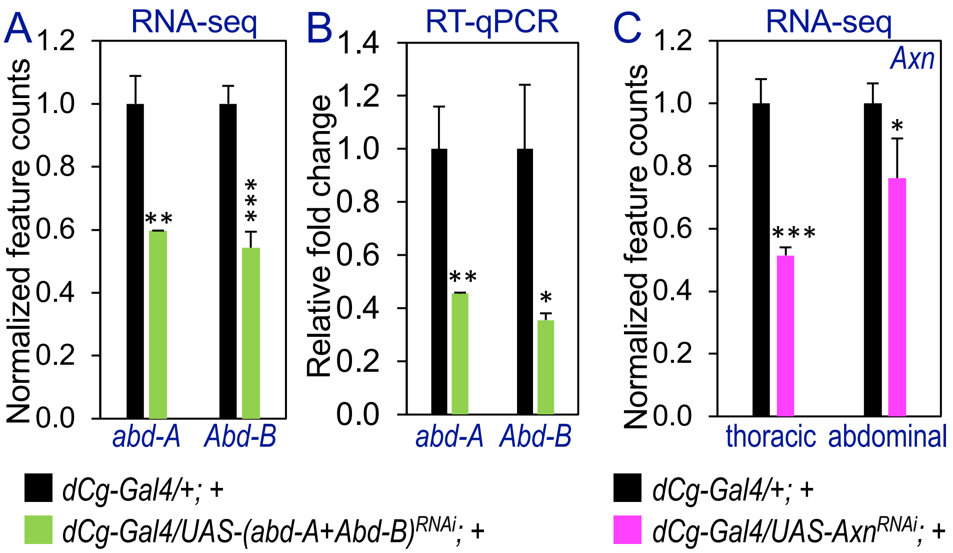

Appendix Figure S11. Bar charts showing mRNA levels of *abd-A*, *Abd-B* and *Axn*. (A, B) Simultaneous depletion of *abd-A* and *Abd-B* in the abdominal fat body results in a 40~60% reduction in their transcript levels, as determined by RNA-seq (A) and RT-qPCR (B). (C) RNA-seq analysis of *Axn* mRNA levels in thoracic and abdominal adipocytes following *Axn* knockdown. Genotypes are color-coded and indicated below each chart (n = 3, independent biological repeats). Statistical significance: $p<0.05$ (*), $p<0.01$ (**), and $p<0.001$ (***); based on one-tailed unpaired *t*-tests because our experimental model is based on well-defined directional predictions.

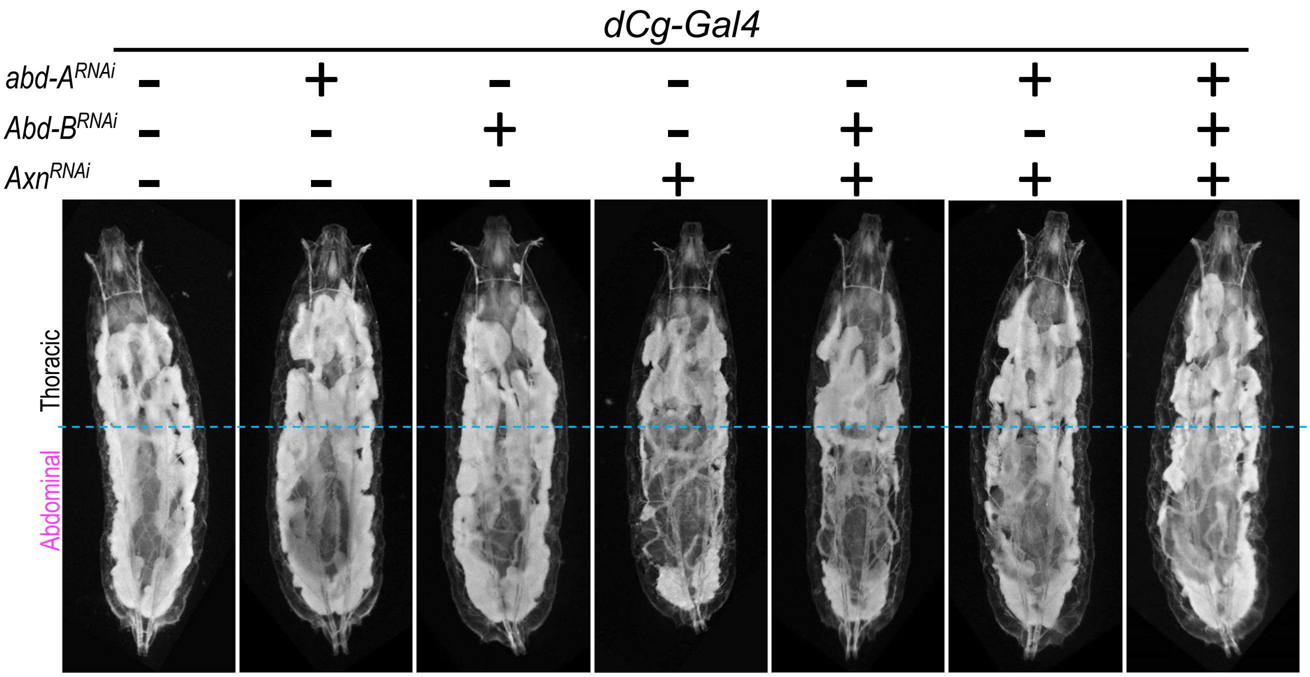

Appendix Figure S12. Whole larvae images for *dCg-Gal4* driven *abd-A* and *Abd-B* depleted larvae with or without active Wnt signaling induced by depleting *Axn.* Note that Wnt signaling-induced fat body defects in the abdominal region of the fat body are significantly rescued by co-depleting *abd-A* and *Abd-B*.

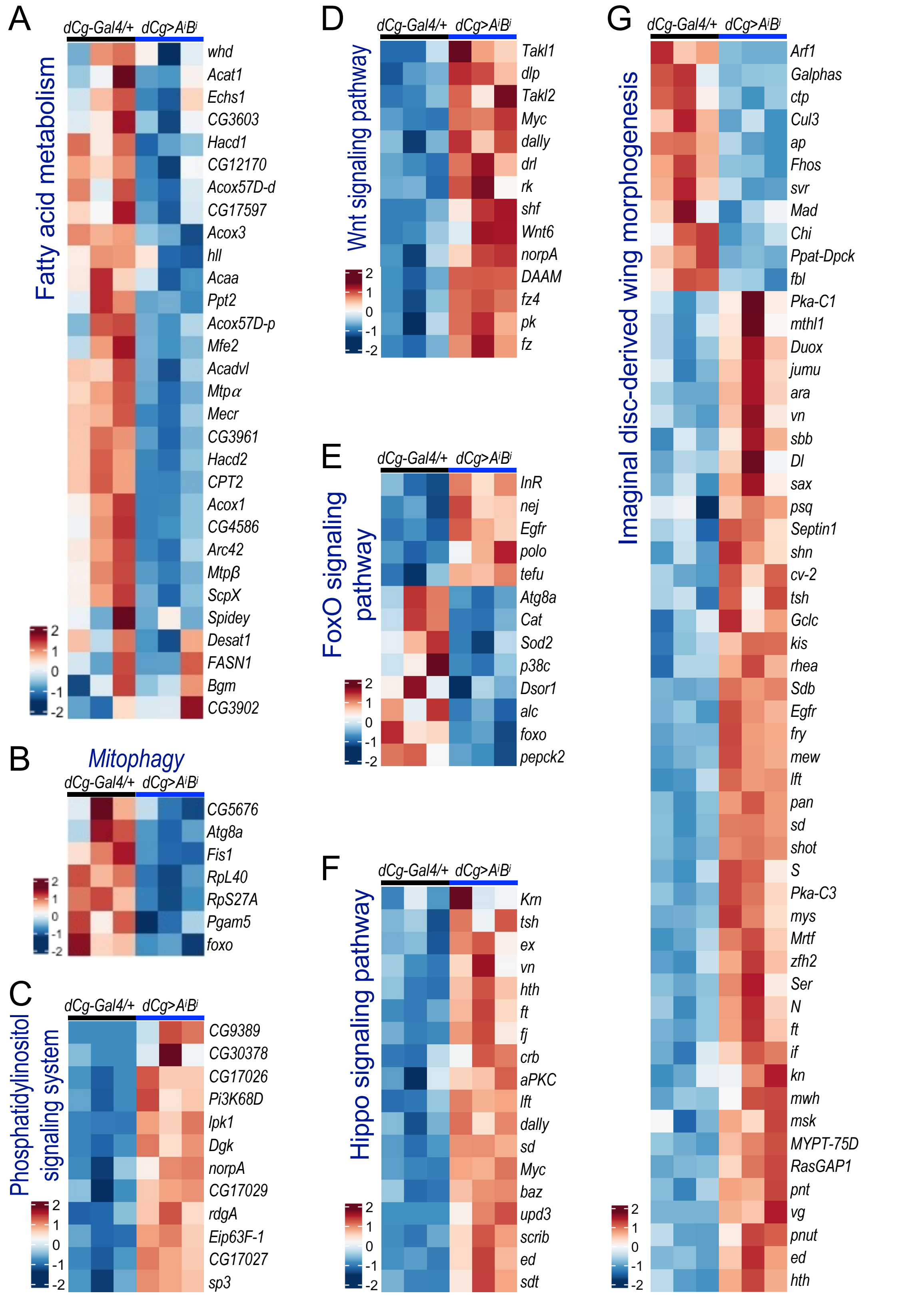

Appendix Figure S13. Effects of Abd-A and Abd-B depletion in larval adipocytes on lipid metabolism and other developmental signaling pathways. Heatmaps show the gene expression levels in various pathways: (A) Fatty acid metabolism, (B) Mitophagy, (C) Phosphatidylinositol signaling system, (D) Wnt signaling pathway, (E) FoxO signaling pathway, (F) Hippo signaling pathway, and (G) Imaginal disc-derived wing morphogenesis (n = 3, independent biological repeats).

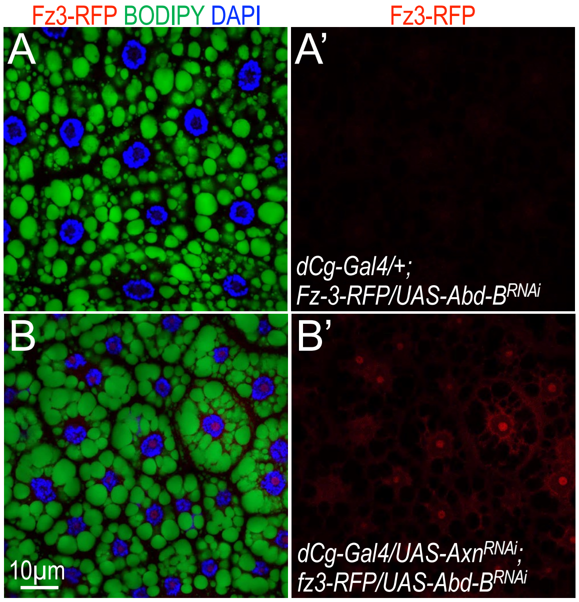

Appendix Figure S14. Abd-B is required for the expression of Wnt-activated target genes in larval adipocytes. Representative confocal images of abdominal adipocytes stained with BODIPY (green) and DAPI (blue), showing *dCg-Gal4* driven depletion of *Abd-B* with or without *Axn* depletion in the *fz3-RFP* background. Genotypes are as follows: (A/A') *dCg-Gal4/+; fz-3-RFP/UAS-Abd-B*^{RNAi}; and (B/B') *dCg-Gal4/UAS-Axn*^{RNAi}*; fz3-RFP/UAS-Abd-B*^{RNAi}. Scale bar in panel (B) applies to all images in this figure: 10 μm.

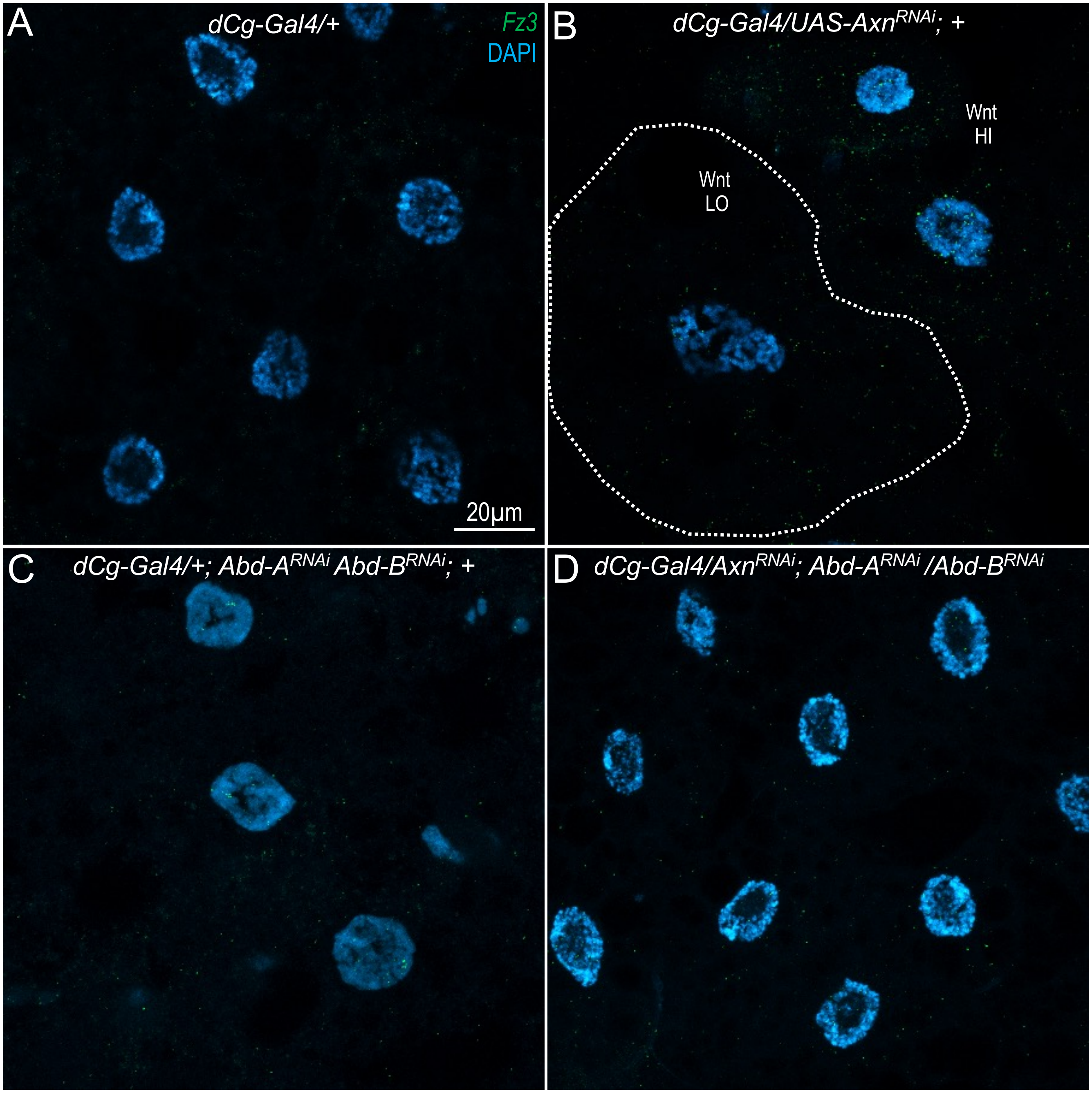

Appendix Figure S15. Detection of mRNA transcripts of *Fz3* in larval adipocytes. *Fz3* mRNA transcripts (green) were visualized using the HCR RNA-FISH assay. DAPI (blue) marks the nuclei positions. Large adipocytes, identified as "Wnt LO" (indicating low Wnt/Wg signaling), are outlined with dotted lines, while adjacent smaller adipocytes are labeled "Wnt HI" (representing high Wnt/Wg signaling). Genotypes: (A) *dCg-Gal4/+; +*, (B) *dCg-Gal4/UAS-Axn^{RNAi}; +*, (C) *dCg-Gal4/+; Abd-A^{RNAi}/Abd-B^{RNAi}*, and (D) *dCg-Gal4/Axn^{RNAi}; Abd-A^{RNAi}/Abd-B^{RNAi}*. The scale bar in panel (A): 20 µm.

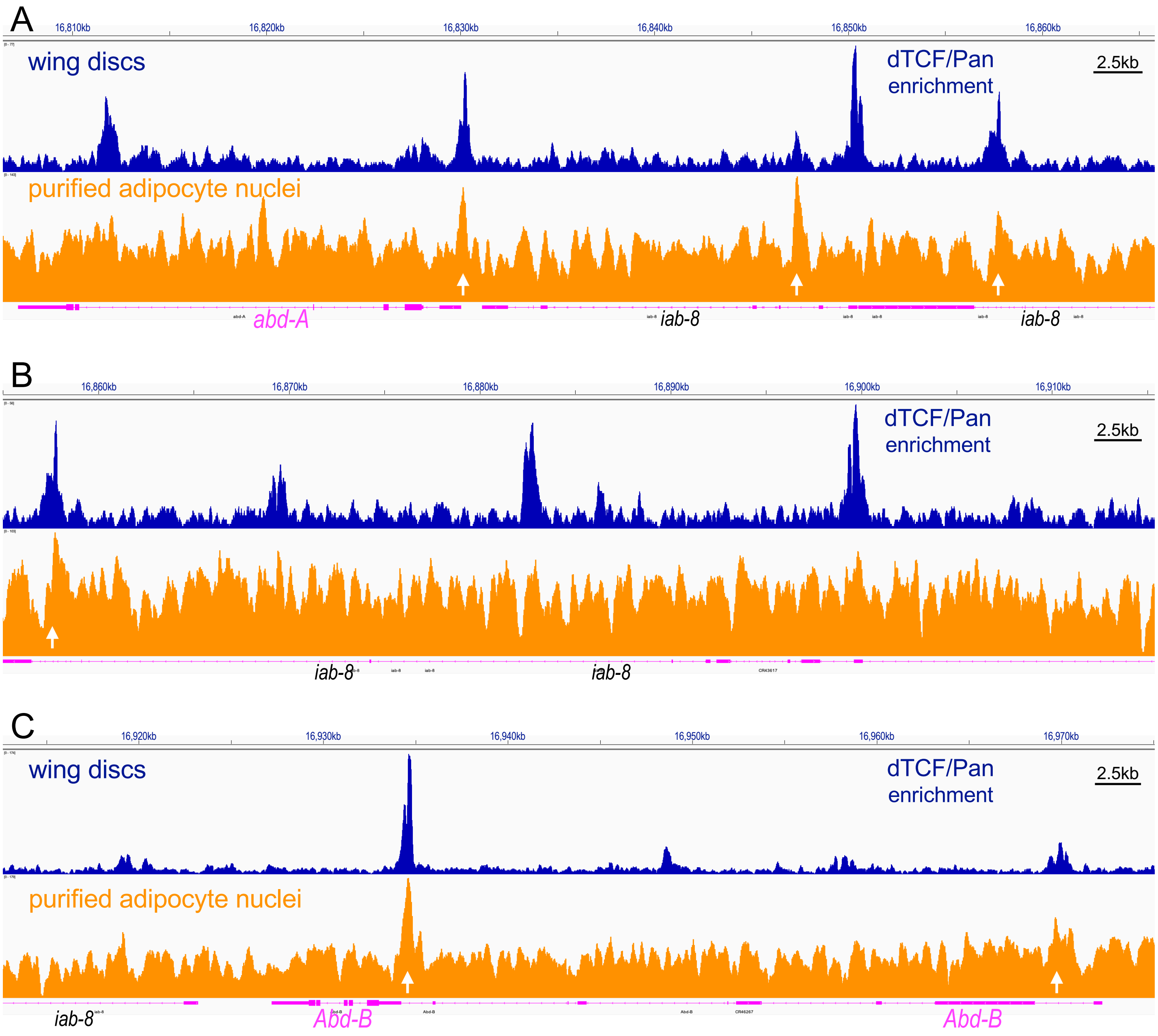

Appendix Figure S16. Genomics tracks showing dTCF/Pan binding peaks at the *abd-A* (A), *iab-8* (B), and *Abd-B* (B) loci. These peaks were identified through CUT&RUN analysis performed on wing discs (dark blue) and purified adipocyte nuclei from the larval fat body (orange). These tracks, visualized using the IGV browser, collectively span the entire *abd-A-Abd-B* genomic region. The y-axis is autoscaled, and different gene isoforms are collapsed and displayed in magenta. Several prominent peaks shared by both datasets are indicated by arrows. Scale bars: 2.5 kb.

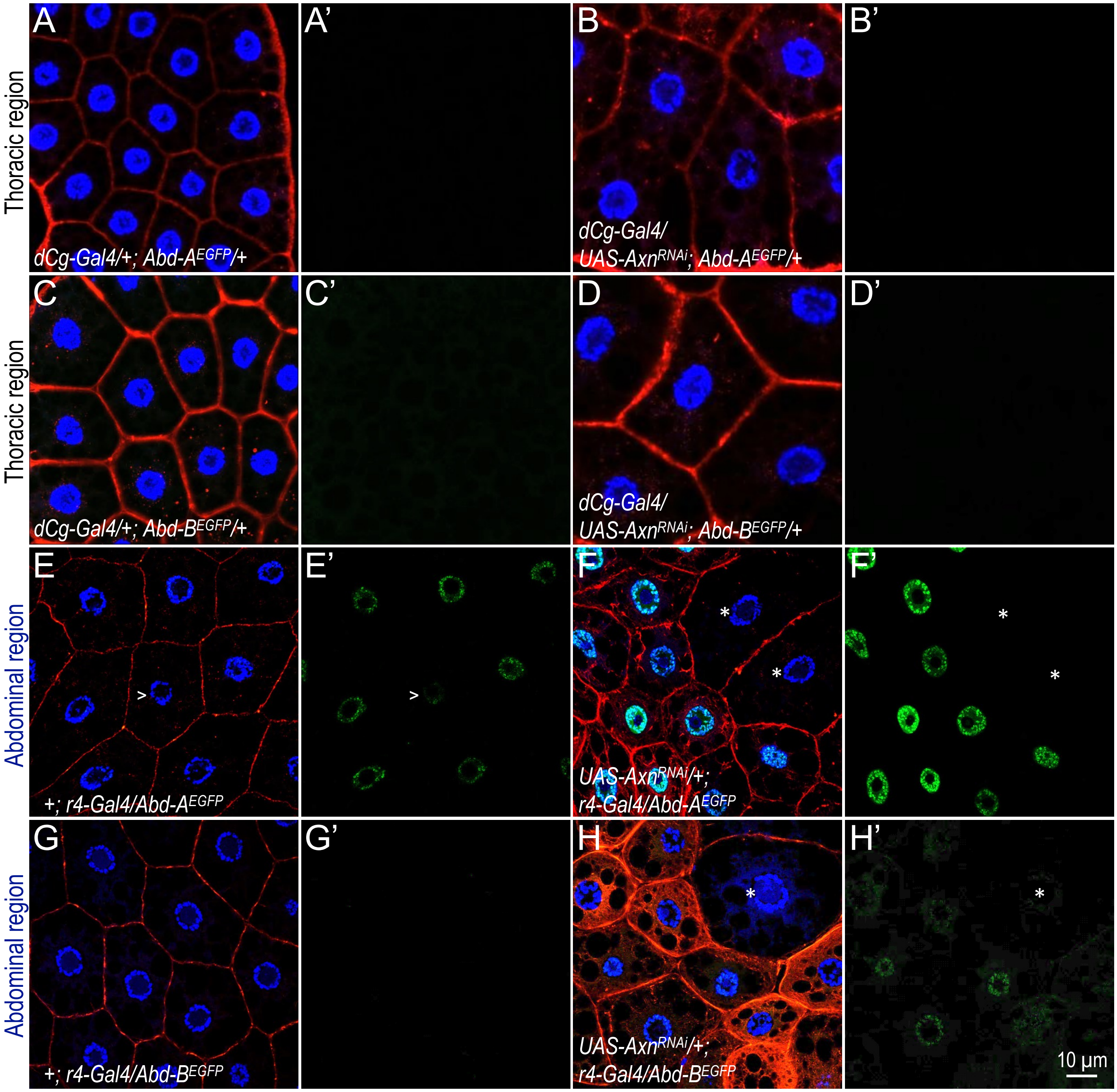

Appendix Figure S17. Effects of Wnt signaling on *abd-A* and *Abd-B* expression in larval adipocytes. (A-B) Thoracic adipocytes stained with DAPI (blue) and Phall (red) in *Abd-A^{EGFP}* background: (A/A') *dCg-Gal4/+; Abd-A^{EGFP}/+*, and (B/B') *dCg-Gal4/UAS-Axn^{RNAi}; Abd-A^{EGFP}/+*. (C-D) Thoracic adipocytes stained with DAPI (blue) and Phall (red) in *Abd-B^{EGFP}* background: (C/C') *dCg-Gal4/+; Abd-B^{EGFP}/+*, and (D/D') *dCg-Gal4/UAS-Axn^{RNAi}; Abd-B^{EGFP}/+*. (E-F) Abdominal adipocytes stained with DAPI (blue) and Phall (red) in *Abd-A^{EGFP}* background: (E/E') +; *r4-Gal4/Abd-A^{EGFP}*, and (F/F') *UAS-Axn^{RNAi}/+; r4-Gal4/Abd-A^{EGFP}*. (G-H) Abdominal adipocytes stained with DAPI (blue) and Phall (red) in *Abd-B^{EGFP}* background: (G/G') +; *r4-Gal4/Abd-B^{EGFP}*, and (H/H') *UAS-Axn^{RNAi}/+; r4-Gal4/Abd-B^{EGFP}*. Adipocytes with lower levels of Abd-A expression are marked with '>' in (E/E'), and adipocytes with low Wnt activity are marked with an asterisk (*) in (F/F') and (H/H'). The scale bar in panel (H'): 10 μm.

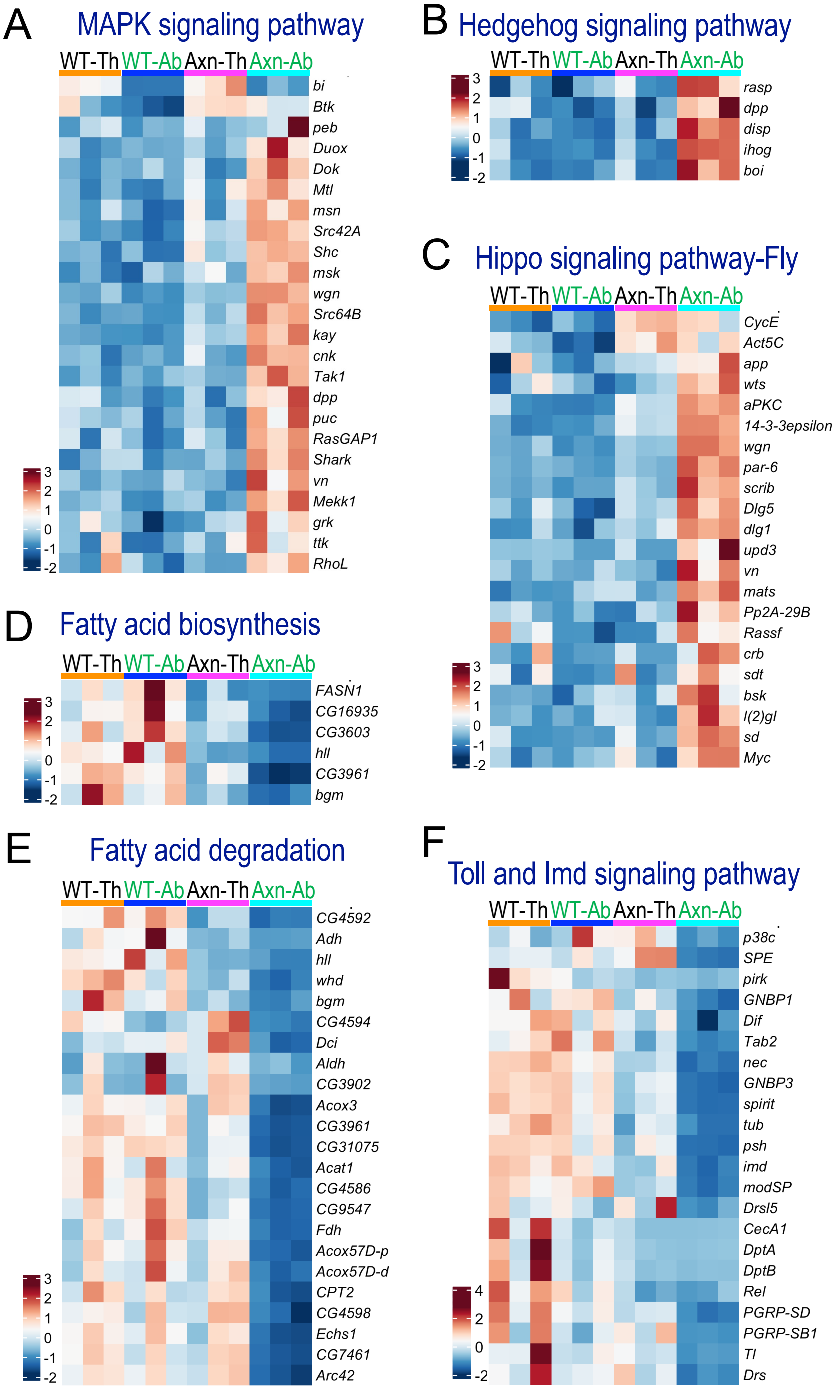

Appendix Figure S18. Heatmaps showing gene expression levels in thoracic and abdominal regions upon activation of Wnt signaling. Thoracic and abdominal regions of '*dCg-Gal4/+;+*' (control) larva are labeled 'WT-Th' and 'WT-Ab', respectively. Thoracic and abdominal regions of '*dCg-Gal4/UAS-Axn*^{RNAi}*; +*' larvae are labeled as 'Axn-Th' and 'Axn-Ab', respectively. Heatmaps display mRNA levels of genes related to: (A) MAPK signaling pathway, (B) Hedgehog signaling pathway, (C) Hippo signaling pathway-Fly, (D) Fatty acid biosynthesis, (E) Fatty acid degradation, and (F) Toll and Imd signaling pathway (n = 3, independent biological repeats).

Appendix Table S1. List of the *Drosophila* stocks used in this study.

Stock #	Genotype	Comments
912	*w[1]; P{w[+mC]=UAS-abd-A.G}21.8*	*UAS-abd-A*
913	*w[1]; P{w[+mC]=UAS-Abd-B.m.C}1.1*	*UAS-Abd-B*
7011	*w[1118]; P{w[+mC]=Cg-GAL4.A}2*	*dCg-Gal4*
7072	*y[1] w[1118]; P{w[+mC]=UAS-drm.G}6.1*	*UAS-Drm*
9902	*w[*]; P{w[+mC]=UAS-odd.H}E*	*UAS-Odd*
29442	*y[1] v[1]; P{y[+t7.7] v[+t1.8]=TRiP.JF03378}attP2*	*UAS-Wnt4[RNAi]*
33832	*y[1] w*; P{w[+mC]=r4-GAL4}3*	*r4-Gal4 (III)*
33986	*y[1] sc* v[1] sev[21]; P{TRiP.HMS00946}attP2*	*UAS-slmb[RNAi]*
34328	*y[1] sc[*] v[1] sev[21]; P{y[+t7.7] v[+t1.8]=TRiP.HMS01315}attP2/TM3, Sb[1]*	*UAS-Odd[RNAi]*
35644	*y[1] sc[*] v[1] sev[21]; P{y[+t7.7] v[+t1.8]=TRiP.GLV21008}attP2*	*UAS-abdA[RNAi]*
35647	*y[1] sc[*] v[1] sev[21]; P{y[+t7.7] v[+t1.8]=TRiP.GLV21012}attP2*	*UAS-AbdB[RNAi]*
38394	*y[1] w[*]; wg[Sp-1]/CyO; P{w[+mC]=GAL4-dSREBPg.K}A45/TM6B, Tb[+]*	*SREBP-Gal4 (III)*
38395	*y[1] w[*]; P{w[+mC]=GAL4-dSREBPg.K}A39; SREBP[189]/TM6B, Tb[1]*	*SREBP-Gal4 (II)*
38625	*w[1118]; PBac{y[+mDint2] w[+mC]=Abd-B-EGFP.S}VK00037/SM5*	*Abd-B[ectopic-EGFP]*
40848	*y[1] v[1]; P{y[+t7.7] v[+t1.8]=TRiP.HMS02015}attP40/CyO*	*UAS-Pan/dTCF[RNAi]*
42548	*y[1] v[1]; P{y[+t7.7] v[+t1.8]=TRiP.HMJ02120}attP40*	*UAS-Drm[RNAi]*
53342	*y[1] v[1]; P{y[+t7.7] v[+t1.8]=TRiP.HMC03571}attP40*	*UAS-Arr[RNAi]*
62434	*y[1] v[1]; P{TRiP.HMJ23888}attP40/CyO*	*UAS-Axn[RNAi], Balancer changed to CyO,Tb*
68187	*y[1] w[*]; P{y[+t7.7] w[+mC]=sfGFP.FLAG-abd-A.B}attP40*	*Abd-A[ectopic-EGFP]*
80070	*w[*]; P{w[+mC]=UAS-Wnt4.G}4-6-1, P{UAS-Wnt4.G}4-6-2; P{UAS-Wnt4.G}13*	*UAS-Wnt4+* (OE)
	+; +; Axn[127]	generated in a previous study (PMID: 28827348)
	Sp/Cyo; fz3-RFP/TM6B	a kind gift from Dr. Yashi Ahmed
	+; +; [EGFP]Ubx	a kind gift from Dr. Ingrid Lohmann
	+; +; Abd-A^{EGFP}	This study
	+; +; Abd-B^{EGFP}	This study
	+; pNP-[abdA]^{RNAi}*; +*	This study
	+; pNP-[AbdB]RNAi; +	This study
	+; pNP-[Ubx]RNAi; +	This study
	+; pNP-[abdA-AbdB]^{RNAi}*: +*	This study
	+; pNP-[abdA-AbdB-Ubx]^{RNAi}*: +*	This study

Appendix Table S2. Primers used in this study.

Primer Names	Primer Sequence [5' to 3']
Abd-A_pNP 5.1	ctagcagtATGGATATGCTTACAGATTAAtagttatattcaagcataTTAATCTGTAAGCATATCCATgcg
Abd-A_pNP 3.1	aattcgcATGGATATGCTTACAGATTAAtatgcttgaatataactaTTAATCTGTAAGCATATCCATactg
Abd-B_pNP 5.1	ctagcagtCCGCAAGCACATGTTCGCCTAtagttatattcaagcataTAGGCGAACATGTGCTTGCGGgcg
Abd-B_pNP 3.1	aattcgcCCGCAAGCACATGTTCGCCTAtatgcttgaatataactaTAGGCGAACATGTGCTTGCGGactg
Ubx_pNP 5.1	ctagcagtCAGGTAAGAGATACTCAGAATtagttatattcaagcataATTCTGAGTATCTCTTACCTGgcg
Ubx_pNP 3.1	aattcgcCAGGTAAGAGATACTCAGAATtatgcttgaatataactaATTCTGAGTATCTCTTACCTGactg
pNP_Test 5.1	GCTGAGAGCATCAGTTGTGA
pNP_Test 3.1	AATCGTGTGTGATGCCTACC
gRNA_Sense oligo [abd-A]	CTTCGTGACGAATTCGGGGGGGTGG
gRNA_Antisense oligo [abd-A]	AAACCCACCCCCCCGAATTCGTCAC
UPS_Hormology Arm [abd-A] 5.1	AATACAACGCAACCCGAGAC
UPS_Homology Arm [abd-A] 3.1	TGCACATATTTTGCTCTCTGG
DWNS_Homology Arm [abd-A] 5.1	ACCACCCCCCCGAATTCG
DWNS_Homology Arm [abd-A] 3.1	TGAGAAGTTCTATCAATGCACTCCA
Excision [abd-A] 5.1	CCCGACAACCACTACCTGAG
Excision [abd-A] 3.1	GATGAGGAGACGGGTGAGTG
gRNA_Sense oligo [Abd-B]	CTTCGAGATGGTGCTGCTGCATGAC
gRNA_Antisense oligo [Abd-B]	AAACGTCATGCAGCAGCACCATCTC
UPS_Hormology Arm [Abd-B] 5.1	TCGACTGGGAGTGTTTCGTT
UPS_Homology Arm [Abd-B] 3.1	GACGGGCAGGGAGGGAT
DWNS_Homology Arm [abd-A] 5.1	AGCAGCAGCATCTGCAGG
DWNS_Homology Arm [abd-A] 3.1	TTAAATGCGTGACGATTCCT
Excision [Abd-B] 5.1	CCCGACAACCACTACCTGAG
Excision [Abd-B] 3.1	CTGCTGTACGGCGACAAGT

	**RT-qPCR Primers [5' to 3']**
CycD 5.1Q	CCAGAAGGATATTACGCCACC
CycD 3.1Q	TGGGTCTTGCGTACTGATTTG
nkd 5.1Q	CTGCATCCTCAACCCGCTTAT
nkd 3.1Q	CCGCCGAGAATTTTATGCTTC
Notum 5.1Q	TTCTGCTGCTCTTAGCCACGTT
Notum 3.1Q	GGTGTCCCTCAATGGATCCTTC
Rp49 5.1Q	ACAGGCCCAAGATCGTGAAGA
Rp49 3.1Q	CGCACTCTGTTGTCGATACCCT
