# Supplementary figures and images for: Adipocyte heterogeneity regulated by the Bithorax Complex-Wnt signaling crosstalk in Drosophila
Source: EMBO Rep. 2025 Dec 3;27(2):367–86. doi: 10.1038/s44319-025-00625-z (PMC12852179; doi:10.1038/s44319-025-00625-z)

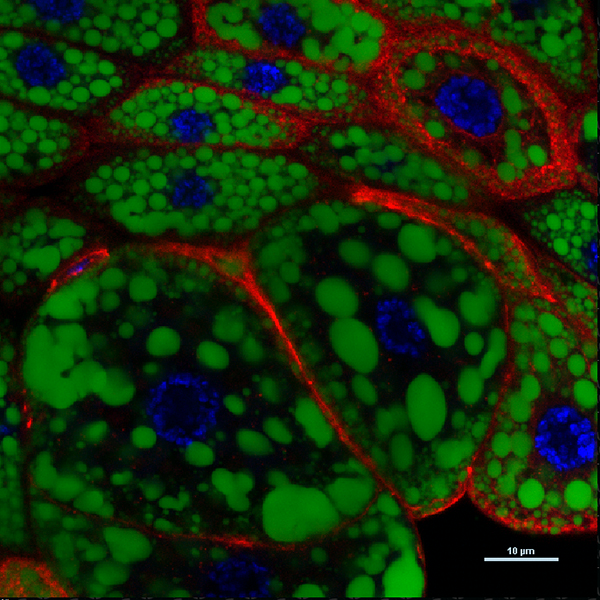

Supplement: Supplementary file 3 — Source data Fig. 1 [file 44319_2025_625_MOESM3_ESM.zip › Figure 1/1H/1H' (SREBP-Gal4>AxnRNAi)_Abdominal.tif]

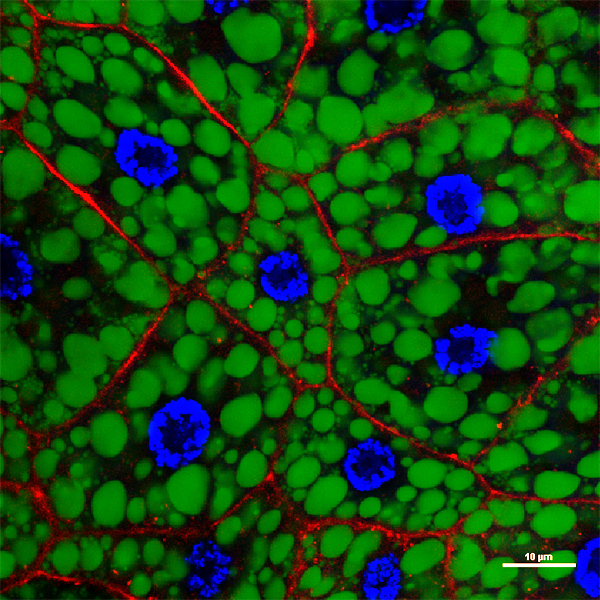

Supplement: Supplementary file 3 — Source data Fig. 1 [file 44319_2025_625_MOESM3_ESM.zip › Figure 1/1H/1H (SREBP-Gal4>AxnRNAi)_Thoracic.tif]

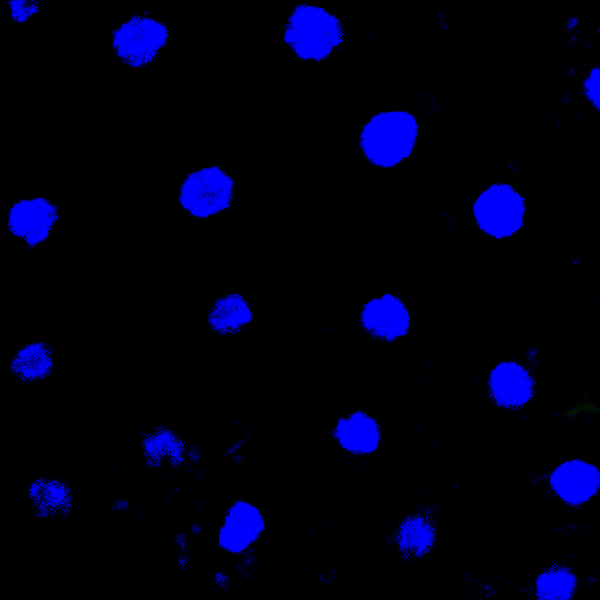

Supplement: Supplementary file 3 — Source data Fig. 1 [file 44319_2025_625_MOESM3_ESM.zip › Figure 1/1O/1O' (Abd-B EGFP-Thoracic)_Green Channel.tif]

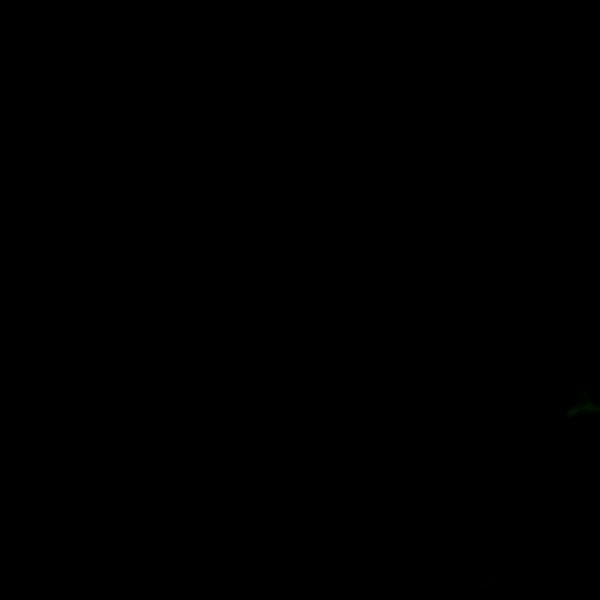

Supplement: Supplementary file 3 — Source data Fig. 1 [file 44319_2025_625_MOESM3_ESM.zip › Figure 1/1O/1O (Abd-B EGFP-Thoracic).tif]

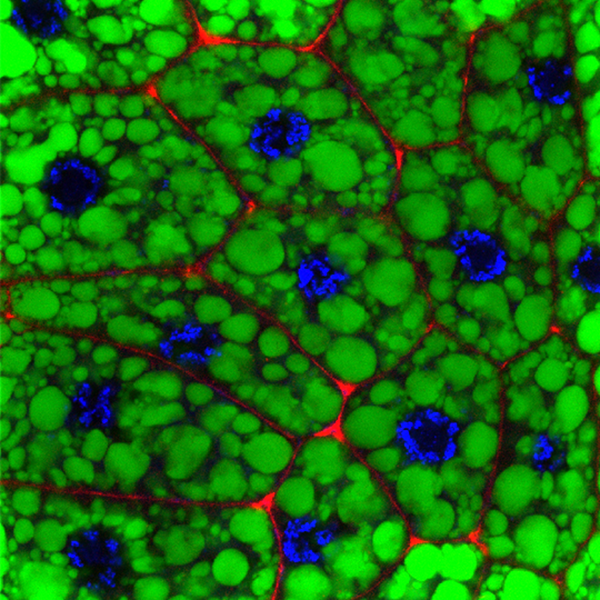

Supplement: Supplementary file 3 — Source data Fig. 1 [file 44319_2025_625_MOESM3_ESM.zip › Figure 1/1F/1F (+; Axn127)_Thoracic.tif]

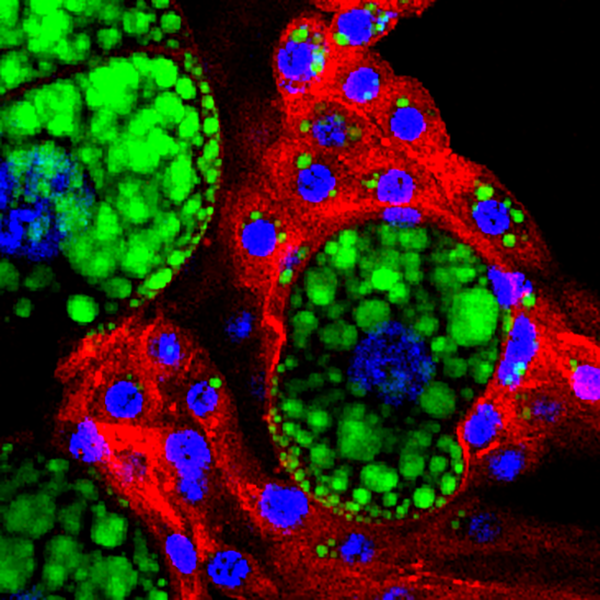

Supplement: Supplementary file 3 — Source data Fig. 1 [file 44319_2025_625_MOESM3_ESM.zip › Figure 1/1F/1F' (+; Axn127)_Abdominal.tif]

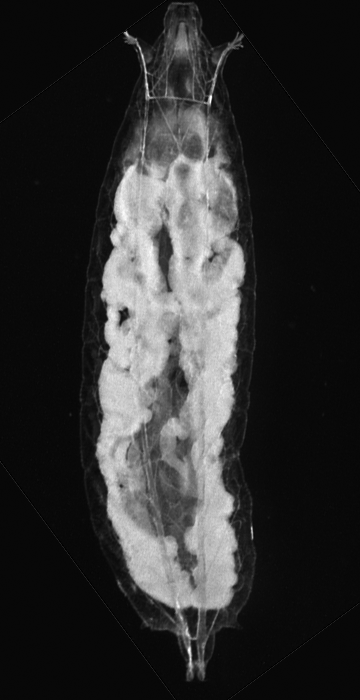

Supplement: Supplementary file 3 — Source data Fig. 1 [file 44319_2025_625_MOESM3_ESM.zip › Figure 1/1A/1A (w1118 larva).tif]

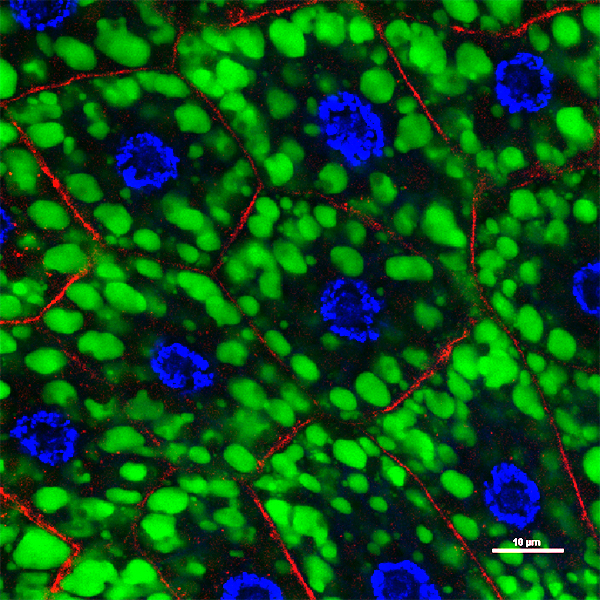

Supplement: Supplementary file 3 — Source data Fig. 1 [file 44319_2025_625_MOESM3_ESM.zip › Figure 1/1G/1G (+; SREBP-Gal4:+)_Thoracic.tif]

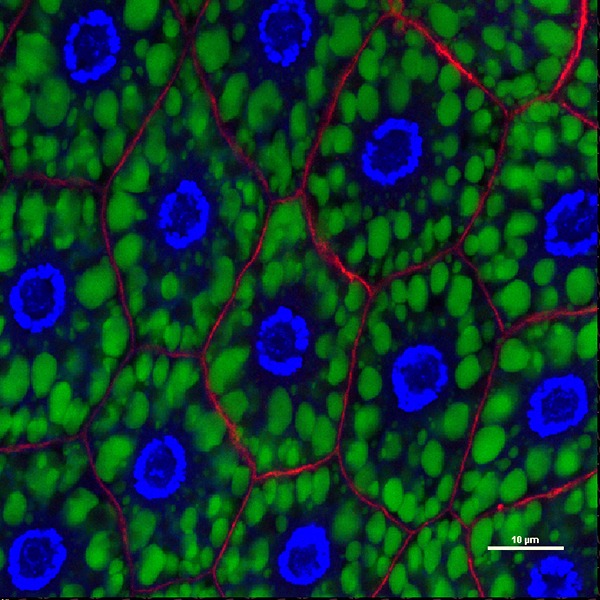

Supplement: Supplementary file 3 — Source data Fig. 1 [file 44319_2025_625_MOESM3_ESM.zip › Figure 1/1G/1G' (+; SREBP-Gal4:+)_Abdominal.tif]

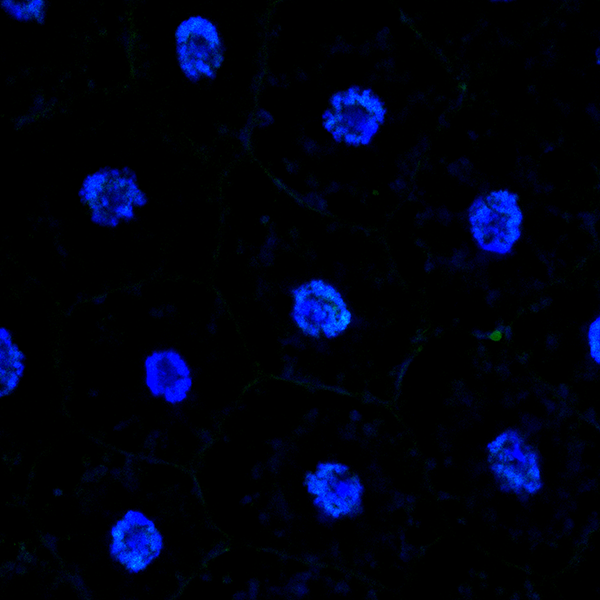

Supplement: Supplementary file 3 — Source data Fig. 1 [file 44319_2025_625_MOESM3_ESM.zip › Figure 1/1N/1N (Abd-A EGFP-Abdominal).tif]

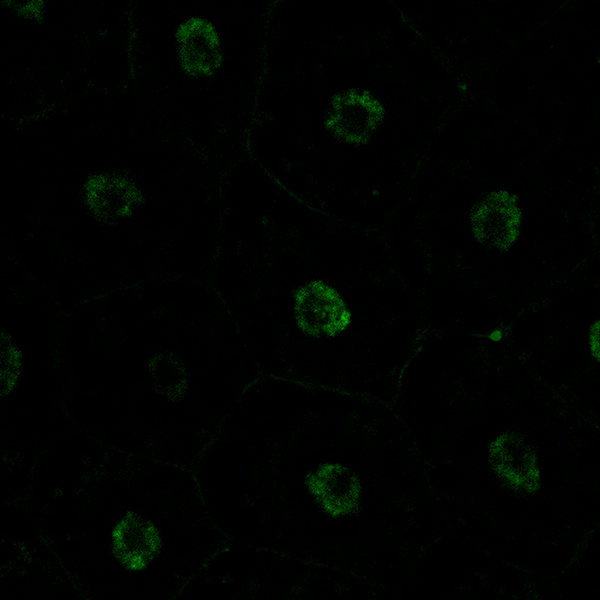

Supplement: Supplementary file 3 — Source data Fig. 1 [file 44319_2025_625_MOESM3_ESM.zip › Figure 1/1N/1N' (Abd-A EGFP-Abdominal)_Green Channel.tif]

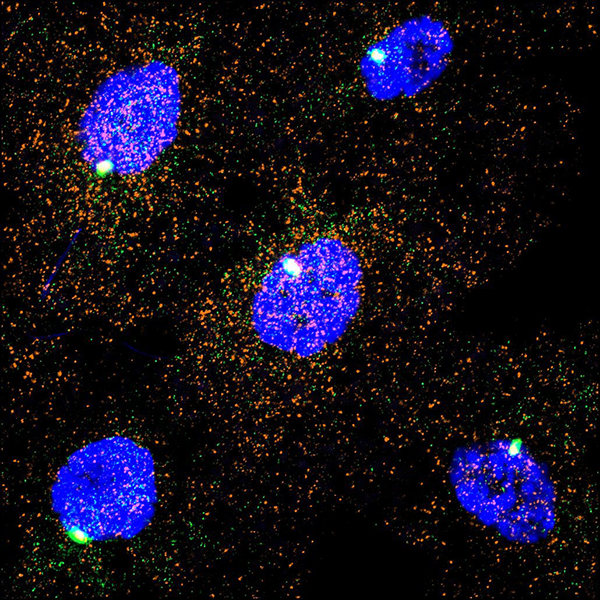

Supplement: Supplementary file 3 — Source data Fig. 1 [file 44319_2025_625_MOESM3_ESM.zip › Figure 1/1L/1L (dCg-Gal4:+; +)_Abdominal.tif]

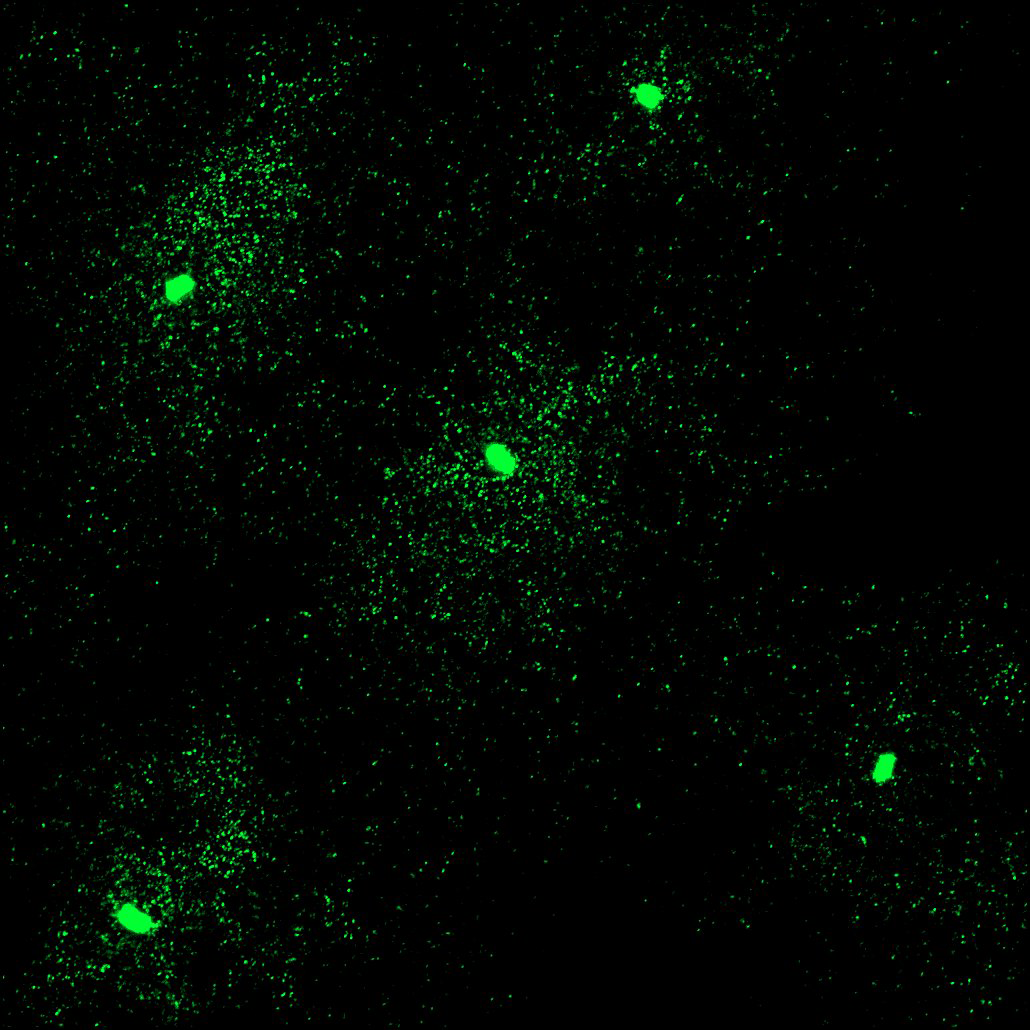

Supplement: Supplementary file 3 — Source data Fig. 1 [file 44319_2025_625_MOESM3_ESM.zip › Figure 1/1L/1L' (dCg-Gal4:+; +)_Abdominal-Green Channel.tif]

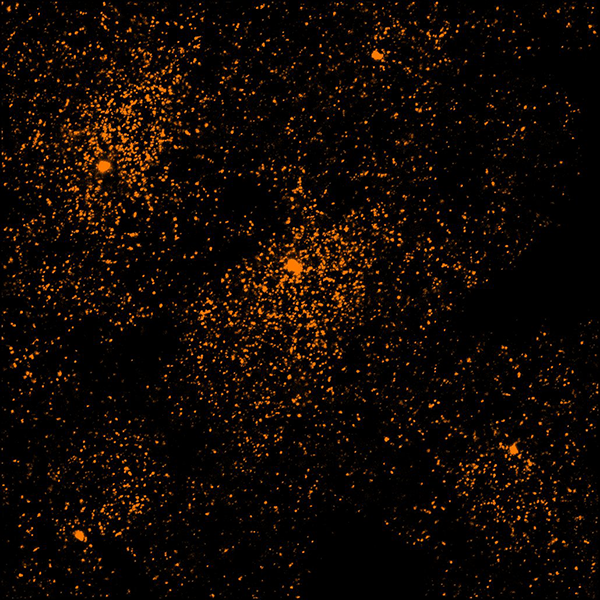

Supplement: Supplementary file 3 — Source data Fig. 1 [file 44319_2025_625_MOESM3_ESM.zip › Figure 1/1L/1L'' (dCg-Gal4:+; +)_Abdominal-Orange Channel.tif]

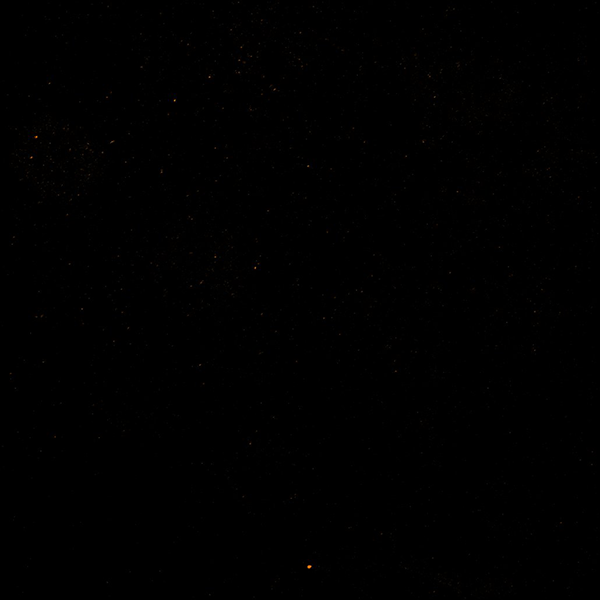

Supplement: Supplementary file 3 — Source data Fig. 1 [file 44319_2025_625_MOESM3_ESM.zip › Figure 1/1K/1K'' (dCg-Gal4:+; +)_Thoracic-Orange Channel.tif]

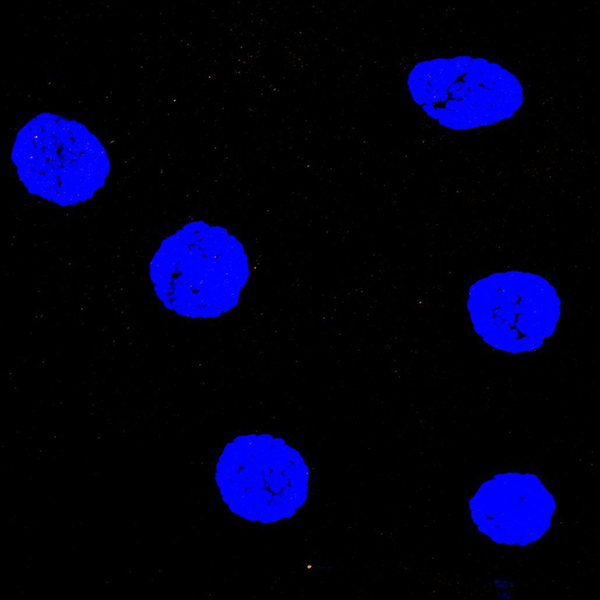

Supplement: Supplementary file 3 — Source data Fig. 1 [file 44319_2025_625_MOESM3_ESM.zip › Figure 1/1K/1K (dCg-Gal4:+; +)_Thoracic.tif]

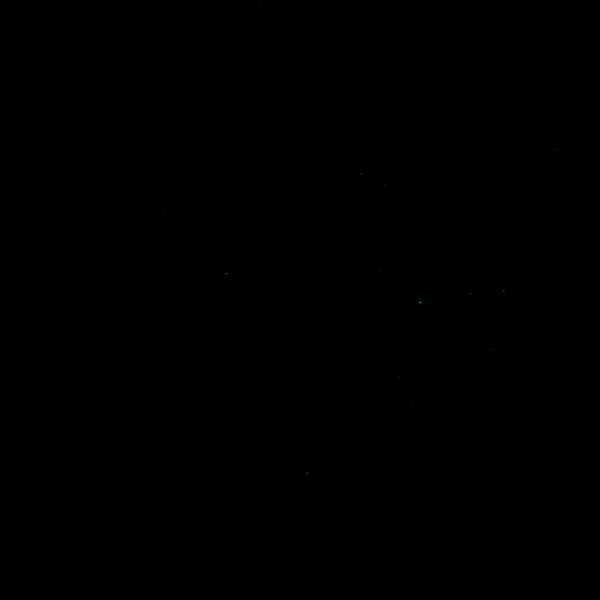

Supplement: Supplementary file 3 — Source data Fig. 1 [file 44319_2025_625_MOESM3_ESM.zip › Figure 1/1K/1K' (dCg-Gal4:+; +)_Thoracic-Green Channel.tif]

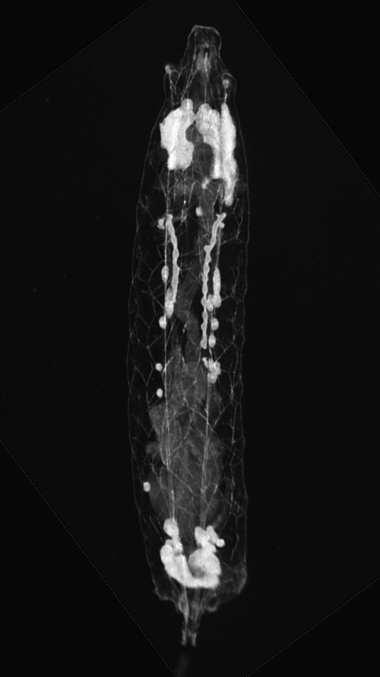

Supplement: Supplementary file 3 — Source data Fig. 1 [file 44319_2025_625_MOESM3_ESM.zip › Figure 1/1B/1B (Axn127 larva).tif]

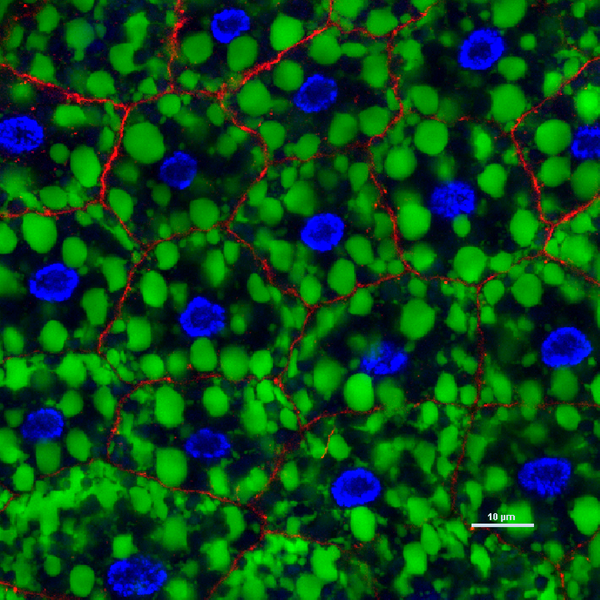

Supplement: Supplementary file 3 — Source data Fig. 1 [file 44319_2025_625_MOESM3_ESM.zip › Figure 1/1E/1E (w1118)_Thoracic.tif]

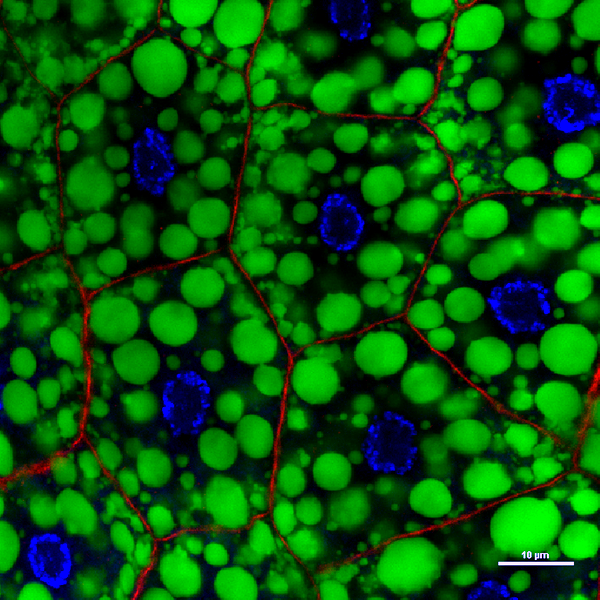

Supplement: Supplementary file 3 — Source data Fig. 1 [file 44319_2025_625_MOESM3_ESM.zip › Figure 1/1E/1E' (w1118)_Abdominal.tif]

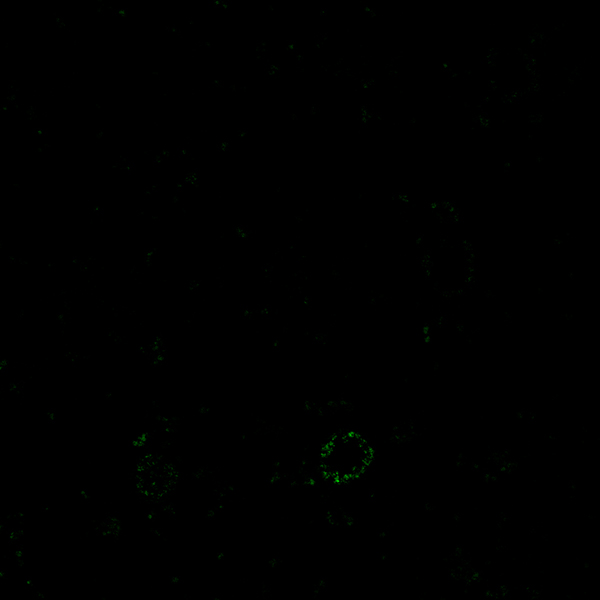

Supplement: Supplementary file 3 — Source data Fig. 1 [file 44319_2025_625_MOESM3_ESM.zip › Figure 1/1P/1P' (Abd-B EGFP-Abdominal)_Green Channel.tif]

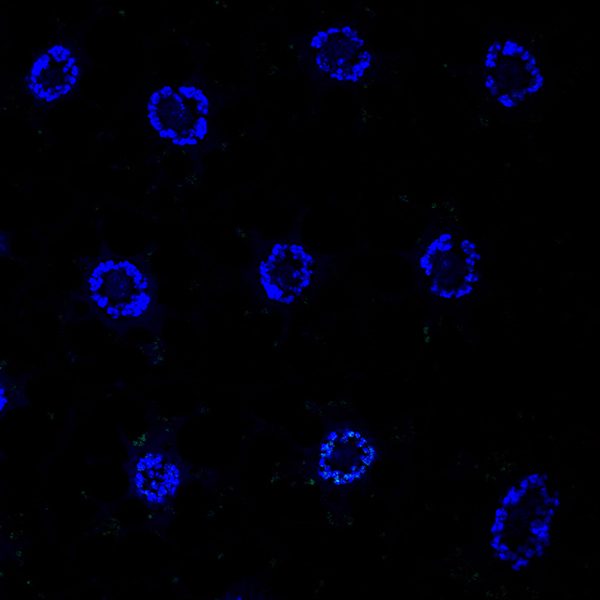

Supplement: Supplementary file 3 — Source data Fig. 1 [file 44319_2025_625_MOESM3_ESM.zip › Figure 1/1P/1P (Abd-B EGFP-Abdominal).tif]

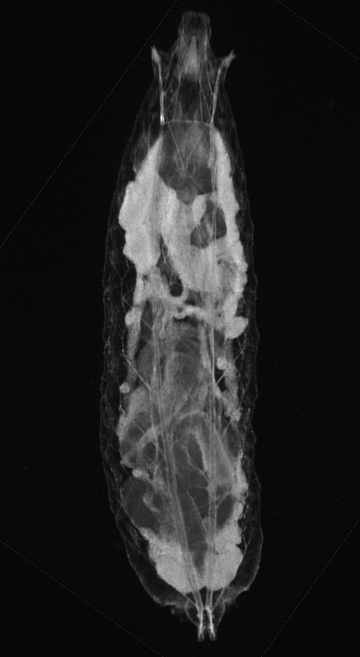

Supplement: Supplementary file 3 — Source data Fig. 1 [file 44319_2025_625_MOESM3_ESM.zip › Figure 1/1D/1D (SREBP-Gal4>AxnRNAi larva).tif]

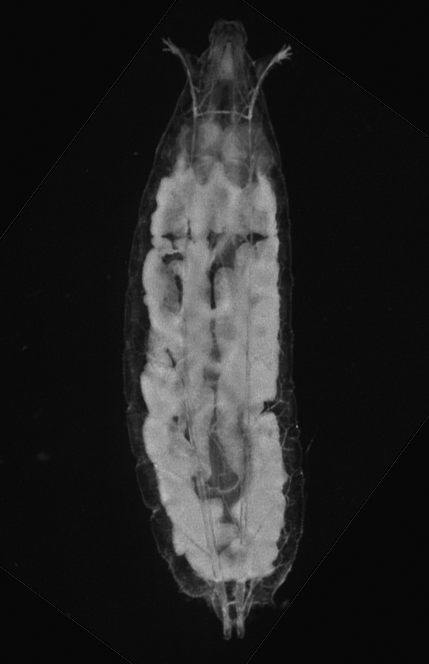

Supplement: Supplementary file 3 — Source data Fig. 1 [file 44319_2025_625_MOESM3_ESM.zip › Figure 1/1C/1C (+; SREBP-Gal4:+ larva).tif]

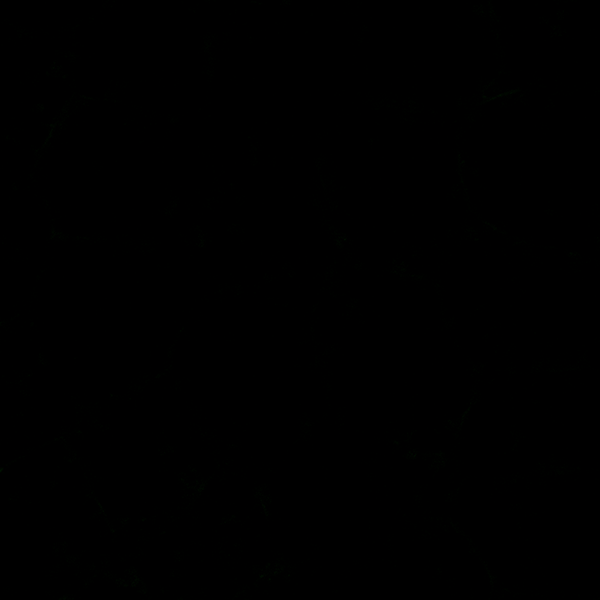

Supplement: Supplementary file 3 — Source data Fig. 1 [file 44319_2025_625_MOESM3_ESM.zip › Figure 1/1M/1M' (Abd-A EGFP-Thoracic)_Green Channel.tif]

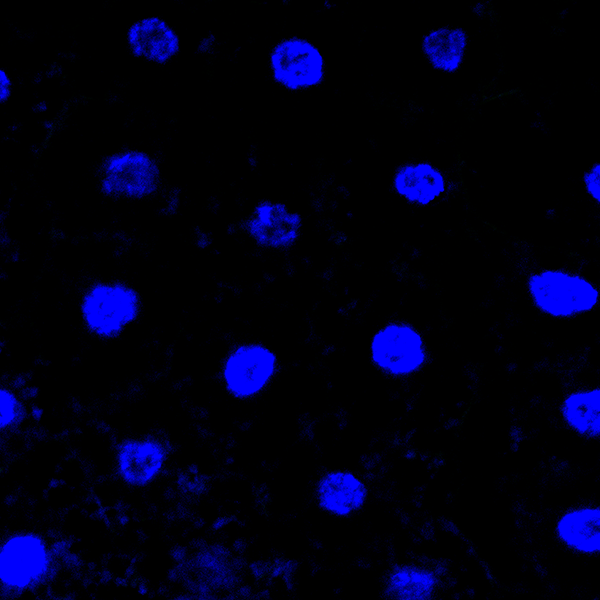

Supplement: Supplementary file 3 — Source data Fig. 1 [file 44319_2025_625_MOESM3_ESM.zip › Figure 1/1M/1M (Abd-A EGFP-Thoracic).tif]

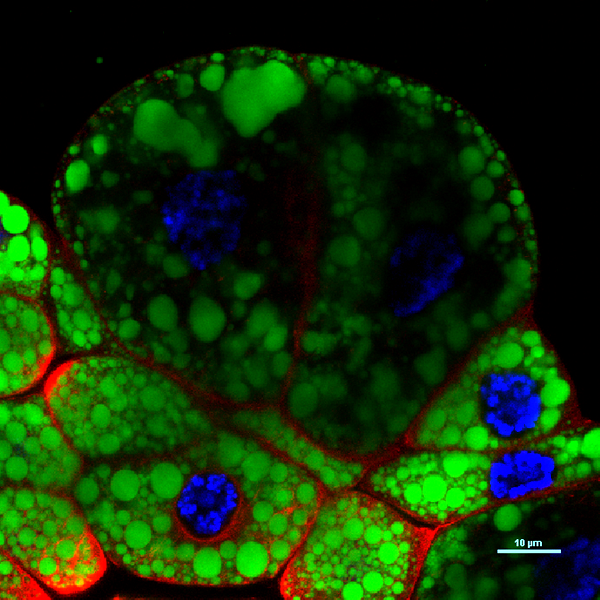

Supplement: Supplementary file 4 — Source data Fig. 2 [file 44319_2025_625_MOESM4_ESM.zip › Figure 2/2G/2G (SREBP>AxnRNAi Abd-B OE)_Thoracic.tif]

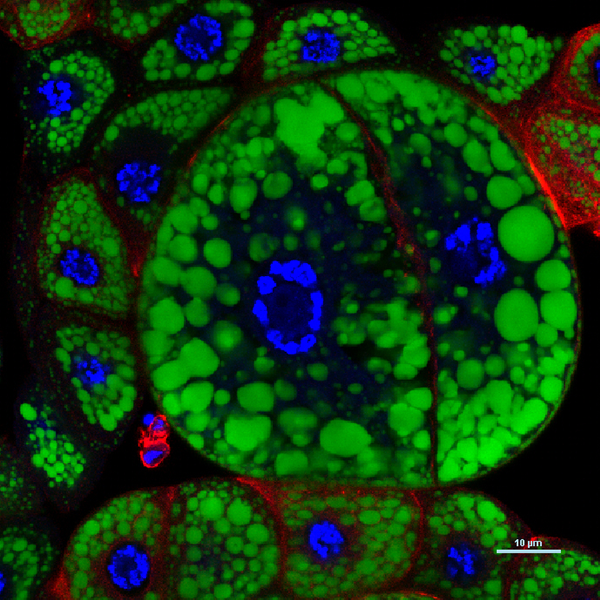

Supplement: Supplementary file 4 — Source data Fig. 2 [file 44319_2025_625_MOESM4_ESM.zip › Figure 2/2G/2G' (SREBP>AxnRNAi Abd-B OE)_Abdominal.tif]

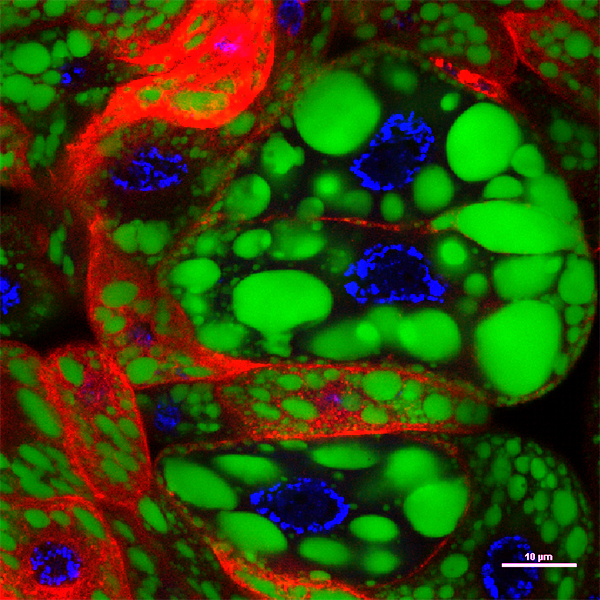

Supplement: Supplementary file 4 — Source data Fig. 2 [file 44319_2025_625_MOESM4_ESM.zip › Figure 2/2I/2I (dCg>AxnRNAi)_Abdominal.tif]

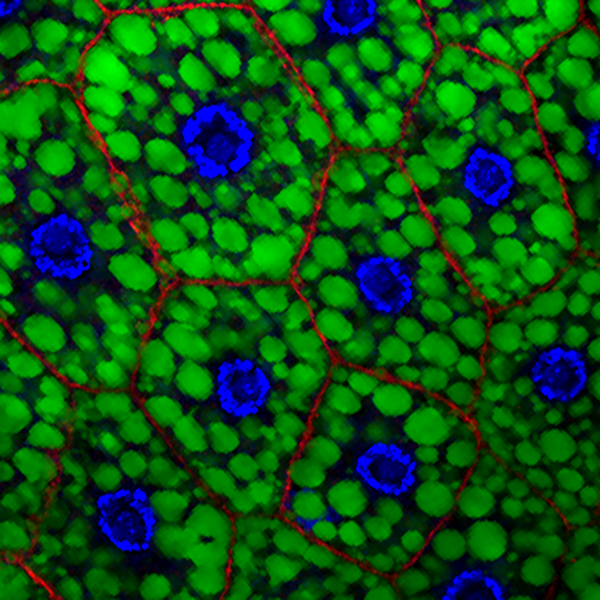

Supplement: Supplementary file 4 — Source data Fig. 2 [file 44319_2025_625_MOESM4_ESM.zip › Figure 2/2N/2N (dCg>abd-ARNAi Abd-BRNAi)-Abdominal.tif]

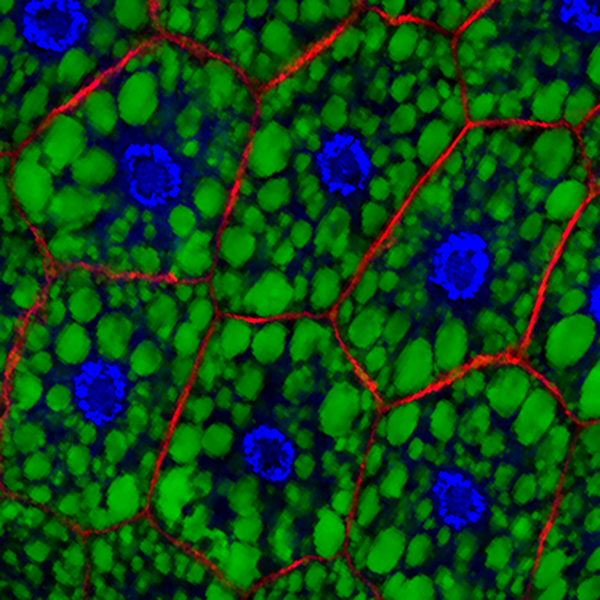

Supplement: Supplementary file 4 — Source data Fig. 2 [file 44319_2025_625_MOESM4_ESM.zip › Figure 2/2O/2O (AxnRNAi abd-ARNAi Abd-BRNAi)-Abdominal.tif]

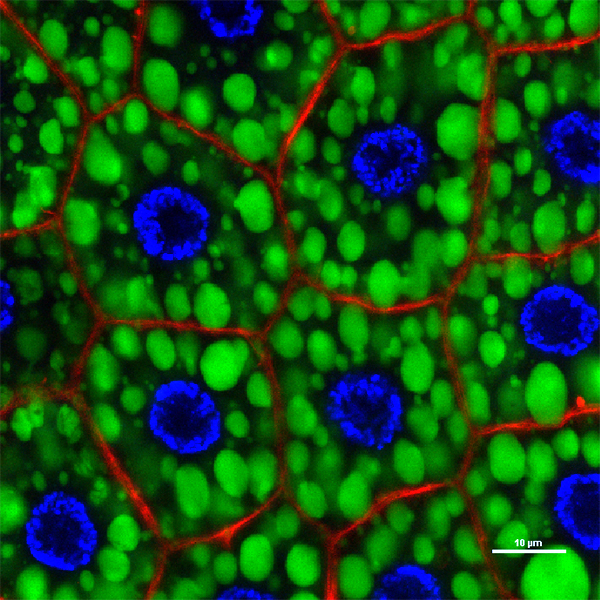

Supplement: Supplementary file 4 — Source data Fig. 2 [file 44319_2025_625_MOESM4_ESM.zip › Figure 2/2H/2H (dCg-Gal4:+)_Abdominal.tif]

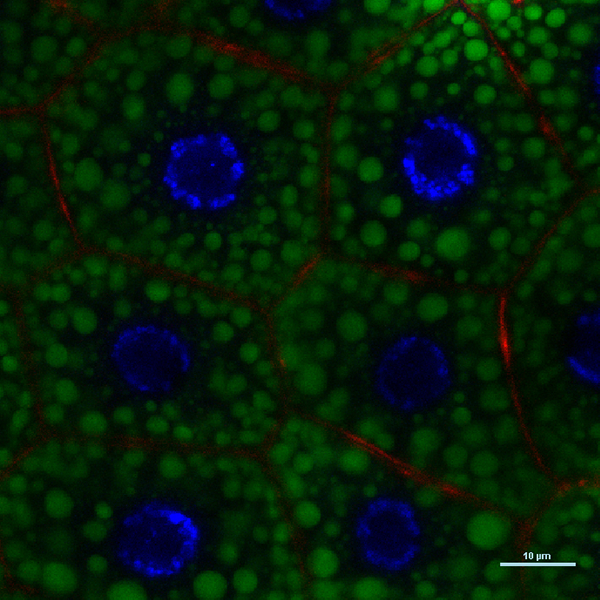

Supplement: Supplementary file 4 — Source data Fig. 2 [file 44319_2025_625_MOESM4_ESM.zip › Figure 2/2F/2F' (SREBP>Abd-B OE)_Abdominal.tif]

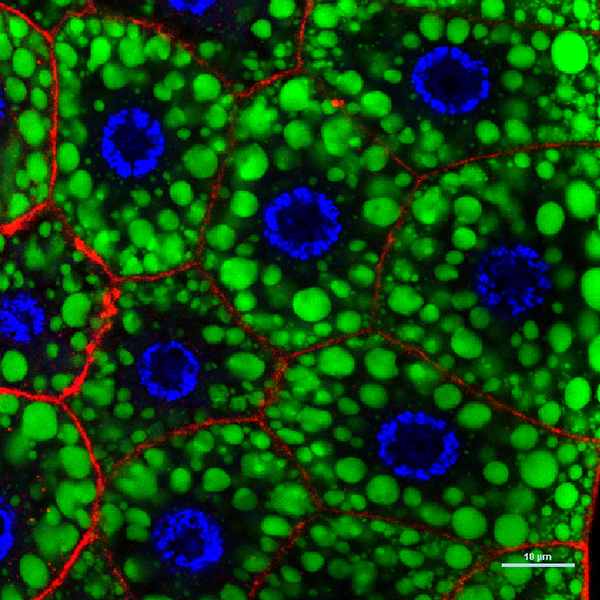

Supplement: Supplementary file 4 — Source data Fig. 2 [file 44319_2025_625_MOESM4_ESM.zip › Figure 2/2F/2F (SREBP>Abd-B OE)_Thoracic.tif]

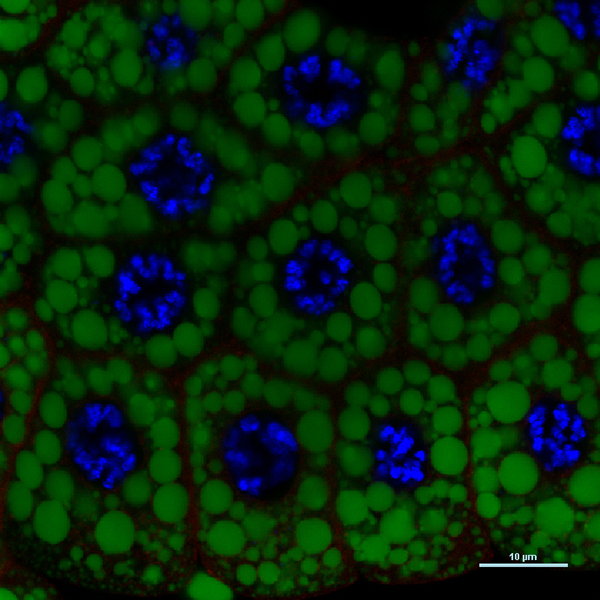

Supplement: Supplementary file 4 — Source data Fig. 2 [file 44319_2025_625_MOESM4_ESM.zip › Figure 2/2C/2C (SREBP>AxnRNAi)_Thoracic.tif]

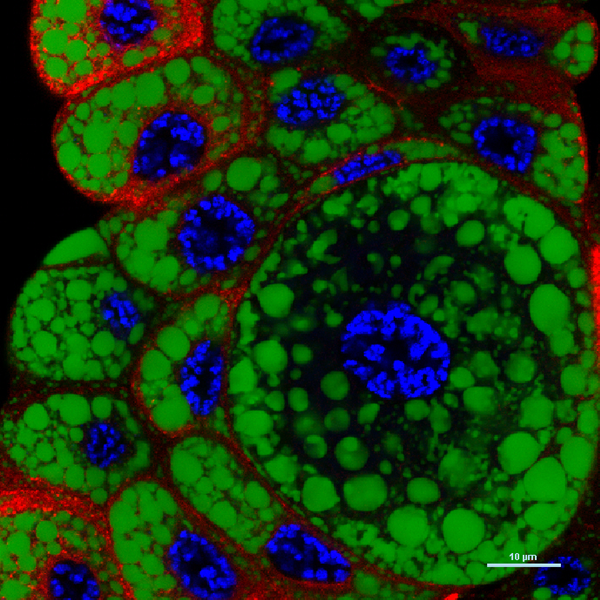

Supplement: Supplementary file 4 — Source data Fig. 2 [file 44319_2025_625_MOESM4_ESM.zip › Figure 2/2C/2C' (SREBP>AxnRNAi)_Abdominal.tif]

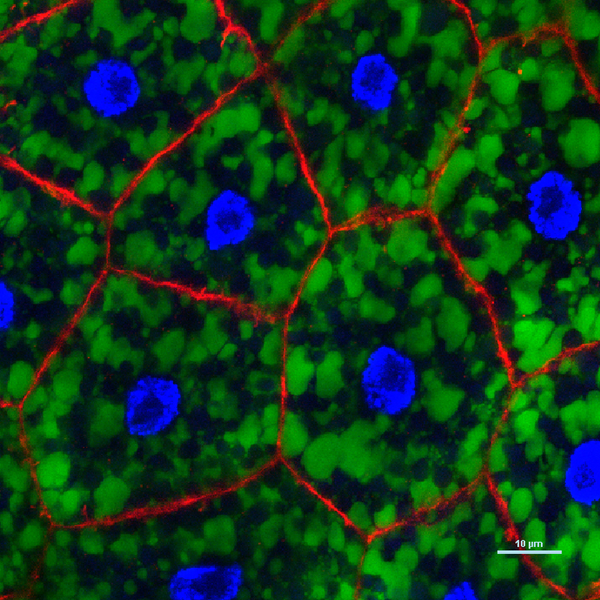

Supplement: Supplementary file 4 — Source data Fig. 2 [file 44319_2025_625_MOESM4_ESM.zip › Figure 2/2D/2D' (SREBP>abd-AOE)_Abdominal.tif]

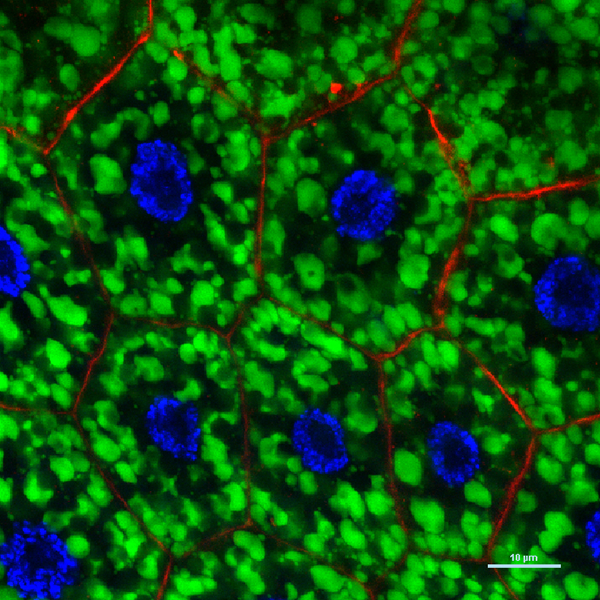

Supplement: Supplementary file 4 — Source data Fig. 2 [file 44319_2025_625_MOESM4_ESM.zip › Figure 2/2D/2D (SREBP>abd-A OE)_Thoracic.tif]

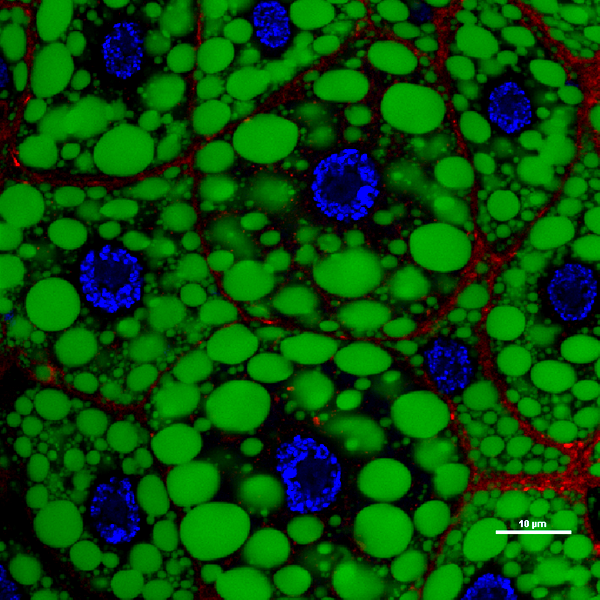

Supplement: Supplementary file 4 — Source data Fig. 2 [file 44319_2025_625_MOESM4_ESM.zip › Figure 2/2M/2M (dCg>AxnRNAi abd-ARNAi)-Abdominal.tif]

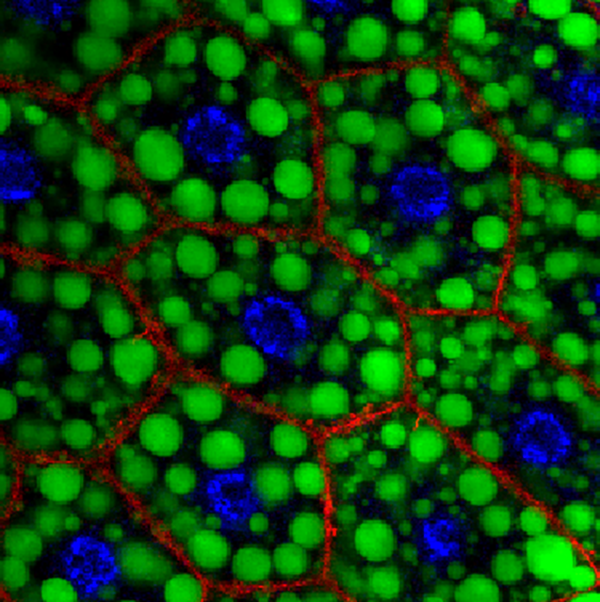

Supplement: Supplementary file 4 — Source data Fig. 2 [file 44319_2025_625_MOESM4_ESM.zip › Figure 2/2J/2J (dCg>Abd-BRNAi)-Abdominal.tif]

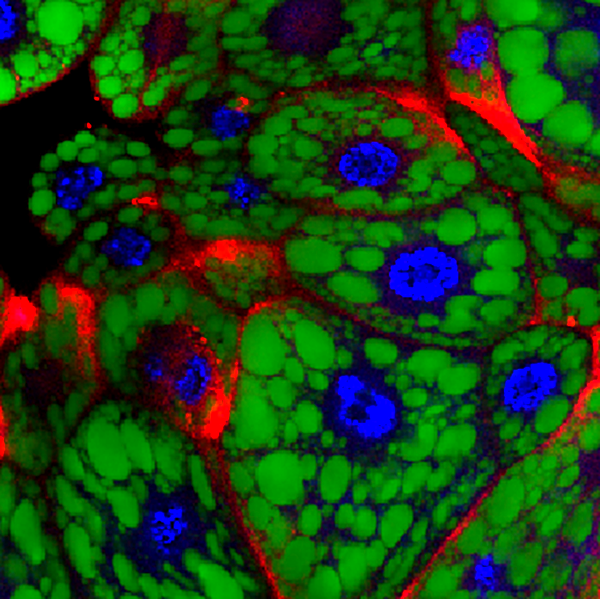

Supplement: Supplementary file 4 — Source data Fig. 2 [file 44319_2025_625_MOESM4_ESM.zip › Figure 2/2K/2K (dCg>AxnRNAi Abd-BRNAi)-Abdominal.tif]

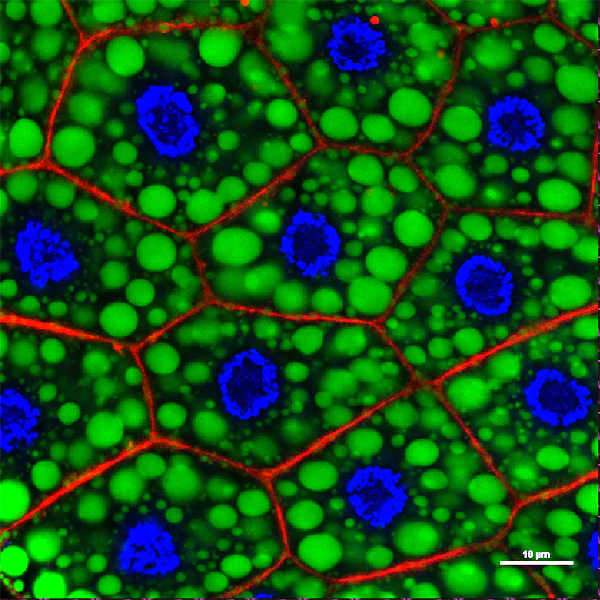

Supplement: Supplementary file 4 — Source data Fig. 2 [file 44319_2025_625_MOESM4_ESM.zip › Figure 2/2L/2L (dCg>abd-ARNAi)-Abdominal.tif]

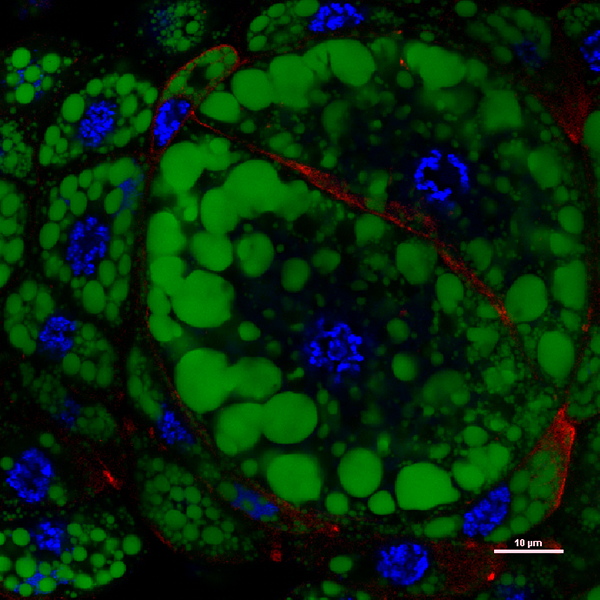

Supplement: Supplementary file 4 — Source data Fig. 2 [file 44319_2025_625_MOESM4_ESM.zip › Figure 2/2E/2E' (SREBP>AxnRNAi abd-A OE)_Abdominal.tif]

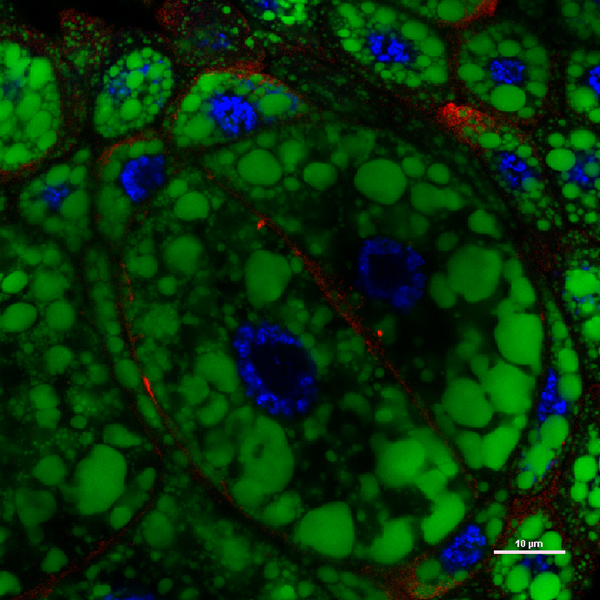

Supplement: Supplementary file 4 — Source data Fig. 2 [file 44319_2025_625_MOESM4_ESM.zip › Figure 2/2E/2E (SREBP>AxnRNAi abd-A OE)_Thoracic.tif]

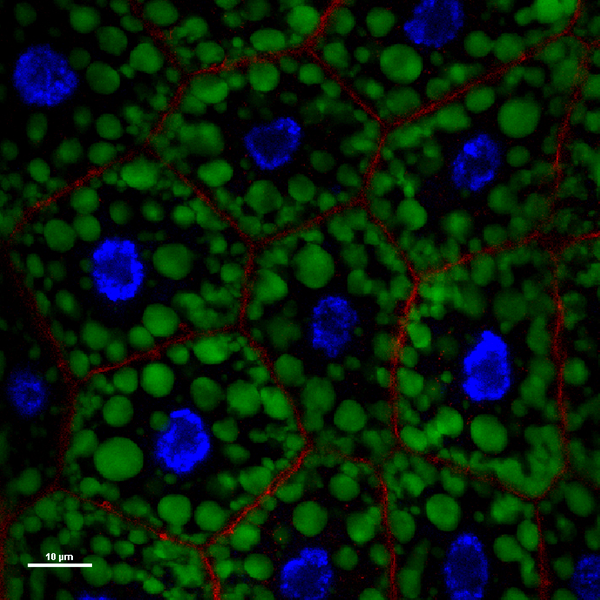

Supplement: Supplementary file 4 — Source data Fig. 2 [file 44319_2025_625_MOESM4_ESM.zip › Figure 2/2B/2B' (SREBP-Gal4:+)_Abdominal.tif]

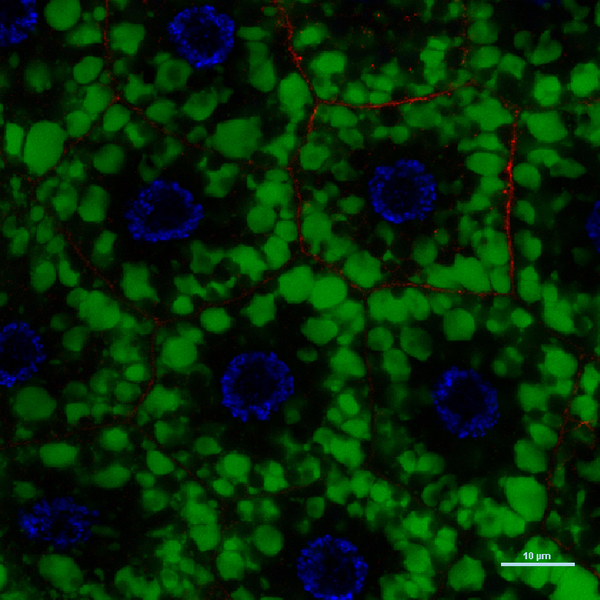

Supplement: Supplementary file 4 — Source data Fig. 2 [file 44319_2025_625_MOESM4_ESM.zip › Figure 2/2B/2B (SREBP-Gal4:+)_Thoracic.tif]

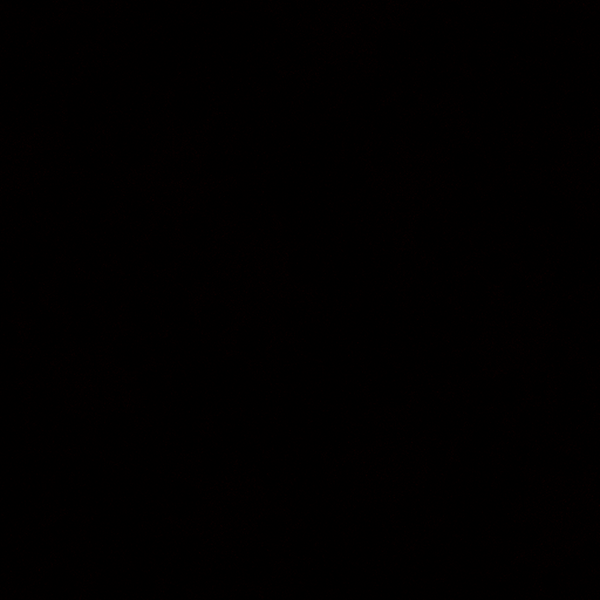

Supplement: Supplementary file 5 — Source data Fig. 4 [file 44319_2025_625_MOESM5_ESM.zip › Figure 4/4E/4E' (dCg-Gal4:+; fz3-RFP:UAS-Abd-ARNAi)_Red Channel.tif]

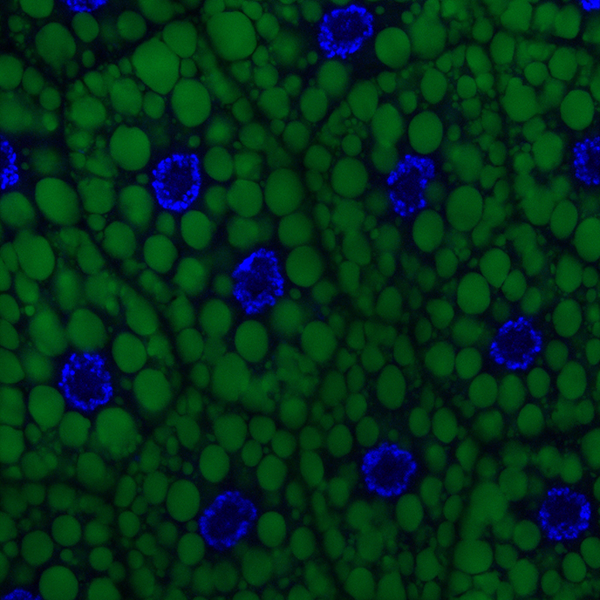

Supplement: Supplementary file 5 — Source data Fig. 4 [file 44319_2025_625_MOESM5_ESM.zip › Figure 4/4E/4E (dCg-Gal4:+; fz3-RFP:UAS-Abd-ARNAi).tif]

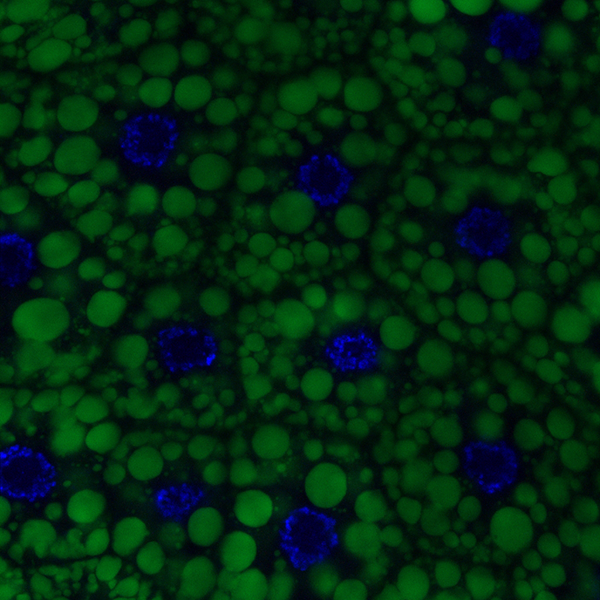

Supplement: Supplementary file 5 — Source data Fig. 4 [file 44319_2025_625_MOESM5_ESM.zip › Figure 4/4C/4C (dCg-Gal4:+; fz3-RFP:+).tif]

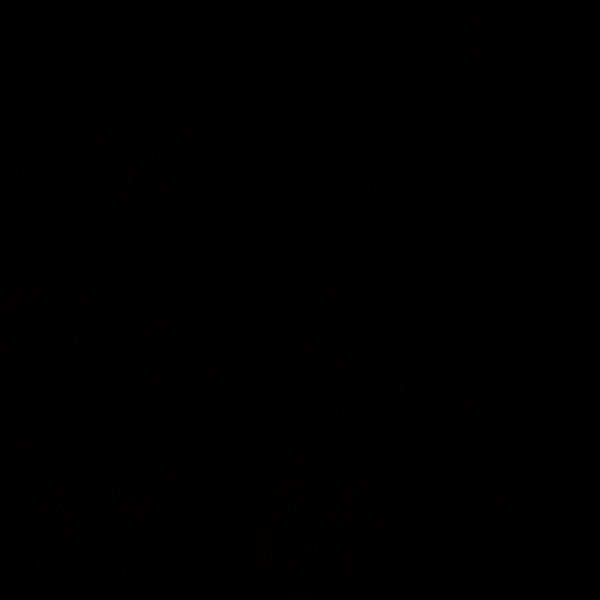

Supplement: Supplementary file 5 — Source data Fig. 4 [file 44319_2025_625_MOESM5_ESM.zip › Figure 4/4C/4C' (dCg-Gal4:+; fz3-RFP:+)_Red Channel.tif]

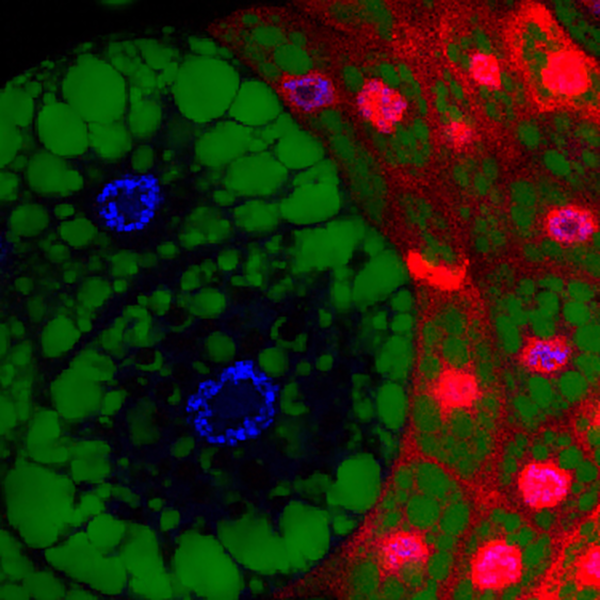

Supplement: Supplementary file 5 — Source data Fig. 4 [file 44319_2025_625_MOESM5_ESM.zip › Figure 4/4D/4D (dCg-Gal4:UAS-AxnRNAi; fz3-RFP:+).tif]

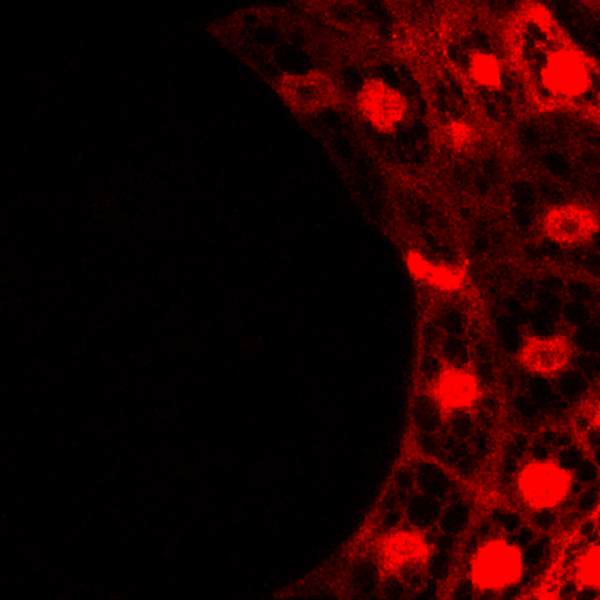

Supplement: Supplementary file 5 — Source data Fig. 4 [file 44319_2025_625_MOESM5_ESM.zip › Figure 4/4D/4D' (dCg-Gal4:UAS-AxnRNAi; fz3-RFP:+)_Red Channel.tif]

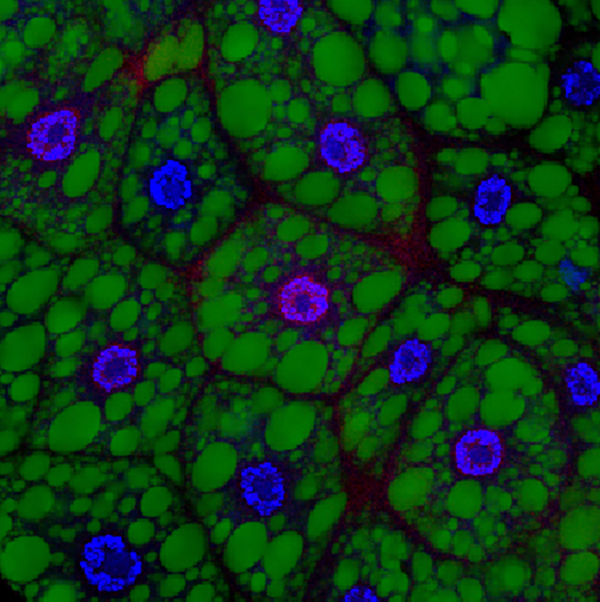

Supplement: Supplementary file 5 — Source data Fig. 4 [file 44319_2025_625_MOESM5_ESM.zip › Figure 4/4F/4F (dCg-Gal4:UAS-AxnRNAi; fz3-RFP:UAS-Abd-ARNAi).tif]

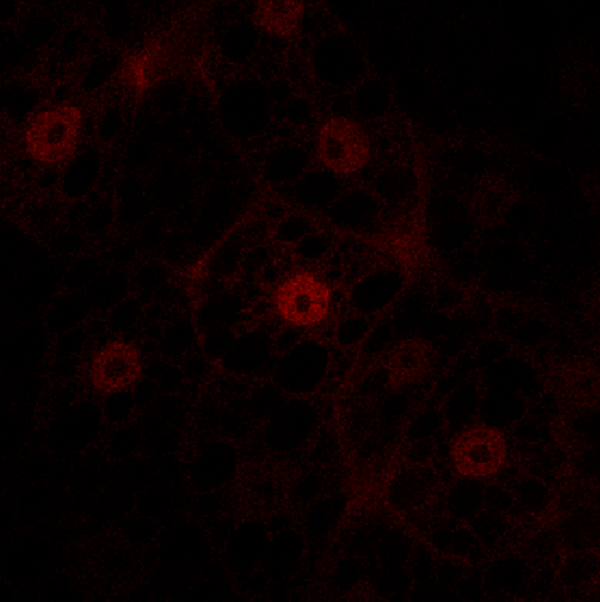

Supplement: Supplementary file 5 — Source data Fig. 4 [file 44319_2025_625_MOESM5_ESM.zip › Figure 4/4F/4F' (dCg-Gal4:UAS-AxnRNAi; fz3-RFP:UAS-Abd-ARNAi)_Red Channel.tif]

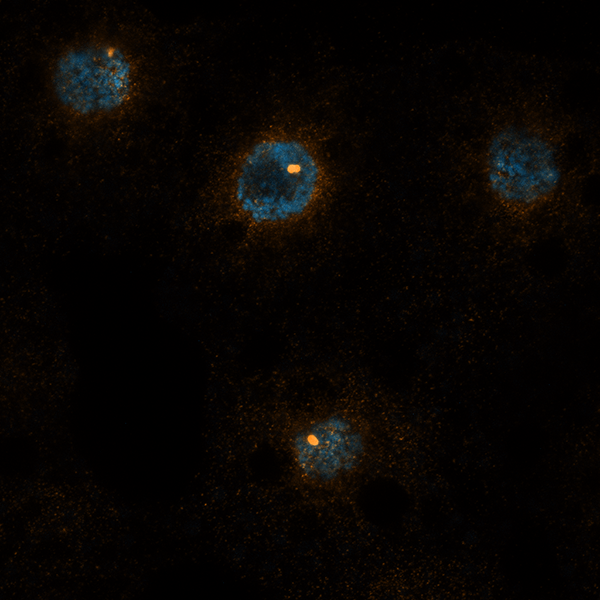

Supplement: Supplementary file 6 — Source data Fig. 5 [file 44319_2025_625_MOESM6_ESM.zip › Figure 5/5G/5G' (AxnRNAi abd-ARNAi Abd-BRNAi)_DAPI & LSD-2.tif]

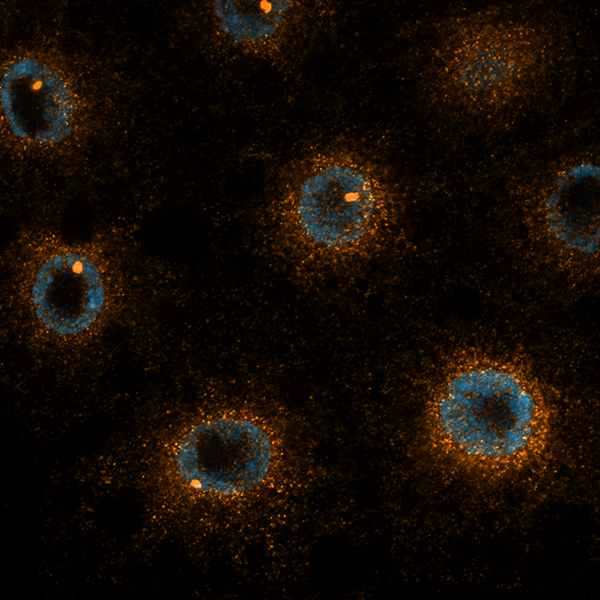

Supplement: Supplementary file 6 — Source data Fig. 5 [file 44319_2025_625_MOESM6_ESM.zip › Figure 5/5F/5F' (abd-ARNAi Abd-BRNAi:+)_DAPI & LSD-2.tif]

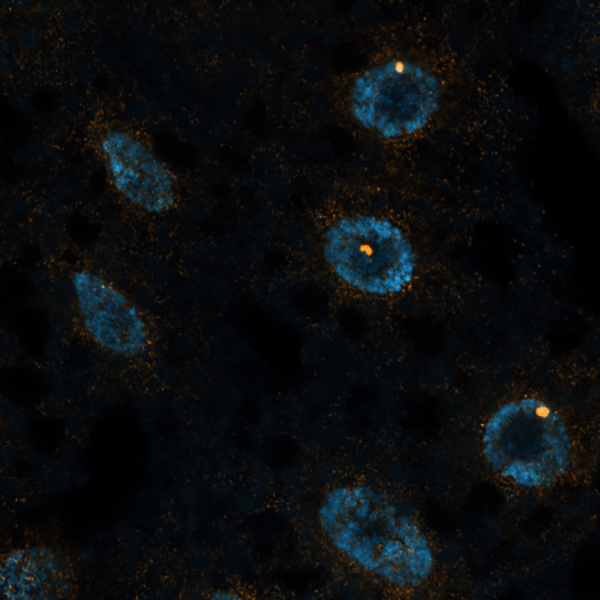

Supplement: Supplementary file 6 — Source data Fig. 5 [file 44319_2025_625_MOESM6_ESM.zip › Figure 5/5D/5D' (dCg-Gal4:+)_DAPI & LSD-2.tif]

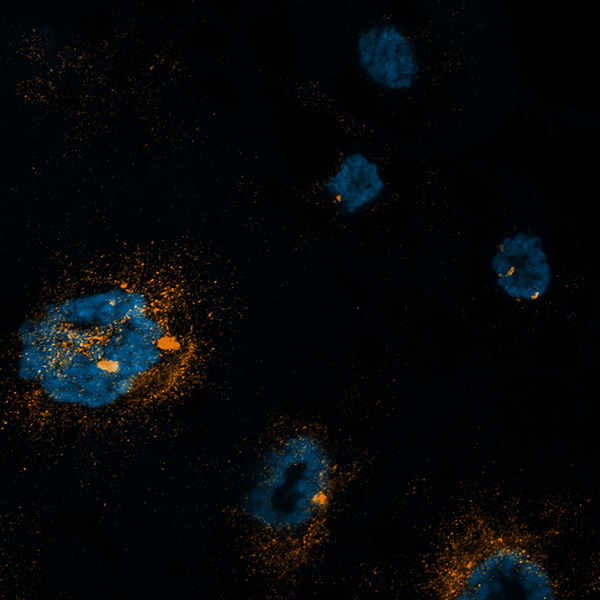

Supplement: Supplementary file 6 — Source data Fig. 5 [file 44319_2025_625_MOESM6_ESM.zip › Figure 5/5E/5E' (dCg-Gal4:UAS-AxnRNAi; +)_DAPI & LSD-2.tif]

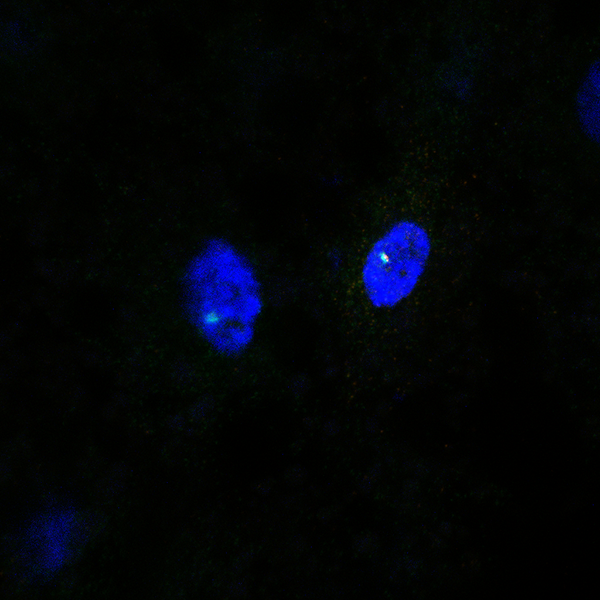

Supplement: Supplementary file 7 — Source data Fig. 6 [file 44319_2025_625_MOESM7_ESM.zip › Figure 6/6F/6F (dCg>arrRNAi;+)-Abdominal_Composite.tif]

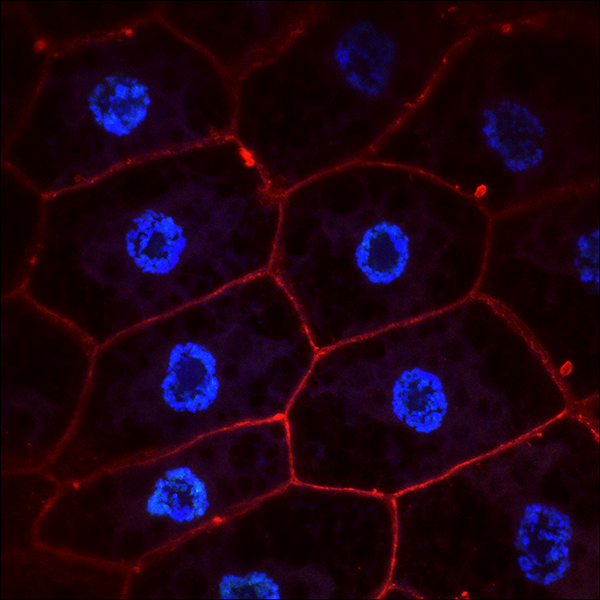

Supplement: Supplementary file 7 — Source data Fig. 6 [file 44319_2025_625_MOESM7_ESM.zip › Figure 6/6H/6H (dCg-Gal4:+; abd-A EGFP:+)-Abdominal.tif]

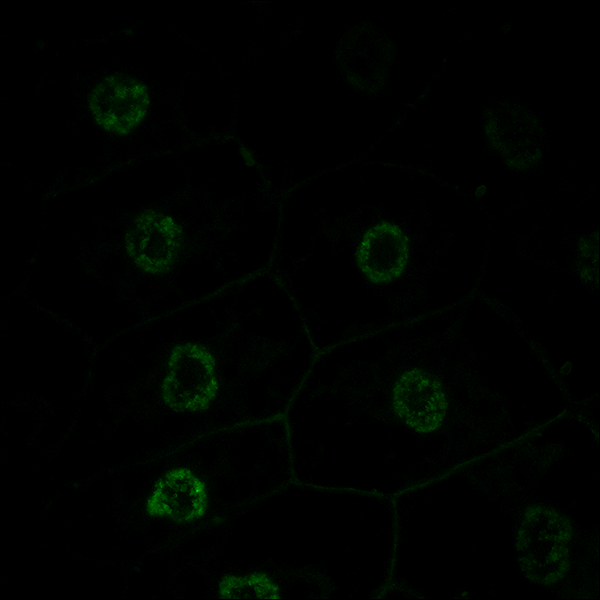

Supplement: Supplementary file 7 — Source data Fig. 6 [file 44319_2025_625_MOESM7_ESM.zip › Figure 6/6H/6H' (dCg-Gal4:+; abd-A EGFP:+)-Abdominal_Green Channel.tif]

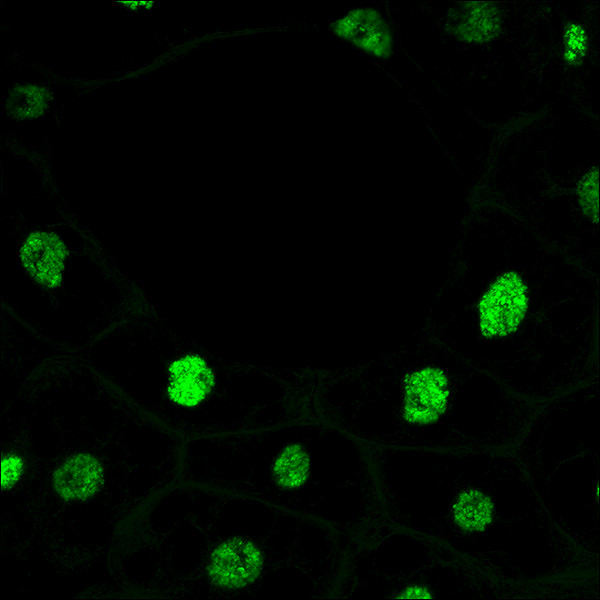

Supplement: Supplementary file 7 — Source data Fig. 6 [file 44319_2025_625_MOESM7_ESM.zip › Figure 6/6I/6I' (dCg>AxnRNAi; abd-A EGFP:+)-Abdominal_Green Channel.tif]

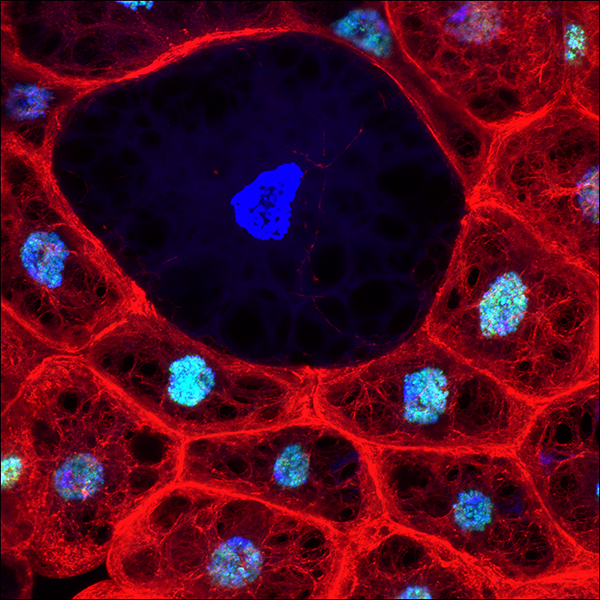

Supplement: Supplementary file 7 — Source data Fig. 6 [file 44319_2025_625_MOESM7_ESM.zip › Figure 6/6I/6I (dCg>AxnRNAi; abd-A EGFP:+)-Abdominal.tif]

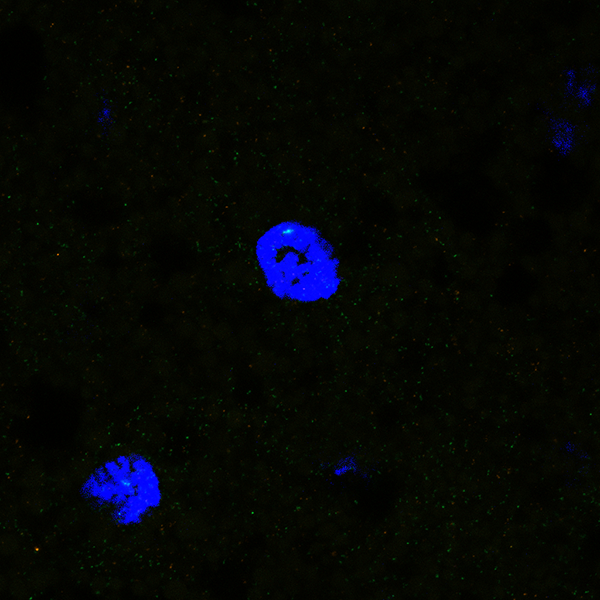

Supplement: Supplementary file 7 — Source data Fig. 6 [file 44319_2025_625_MOESM7_ESM.zip › Figure 6/6G/6G (dCg>dTCFRNAi;+)-Abdominal_Composite.tif]

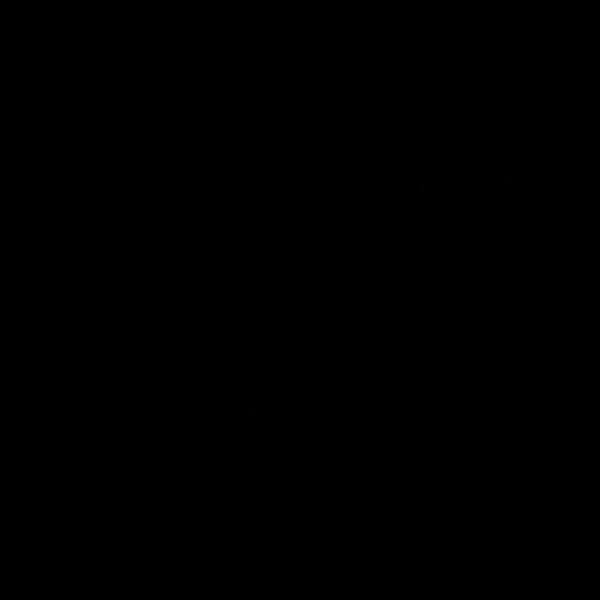

Supplement: Supplementary file 7 — Source data Fig. 6 [file 44319_2025_625_MOESM7_ESM.zip › Figure 6/6B/6B' (dCg-Gal4:+; +)-Thoracic_abdA.tif]

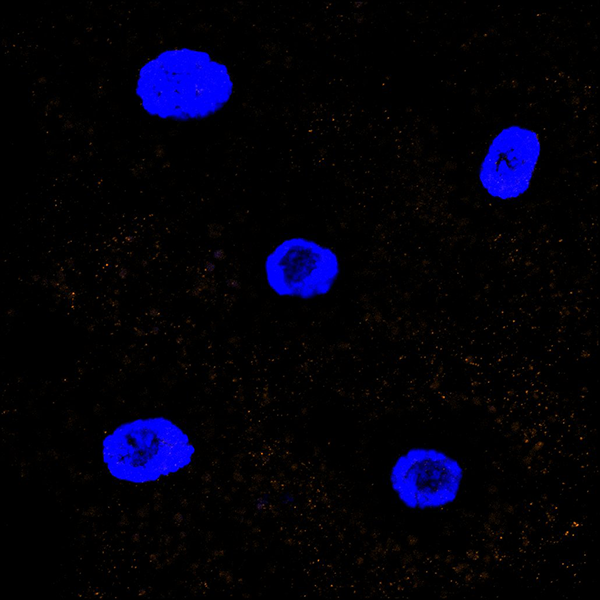

Supplement: Supplementary file 7 — Source data Fig. 6 [file 44319_2025_625_MOESM7_ESM.zip › Figure 6/6B/6B (dCg-Gal4:+; +)-Thoracic_Composite.tif]

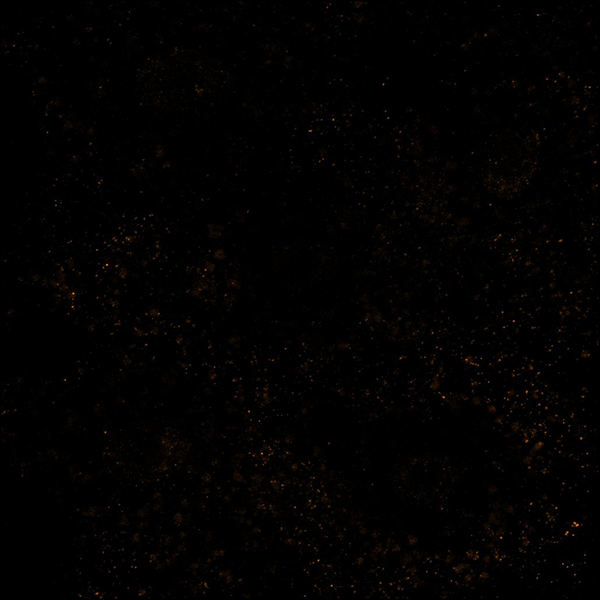

Supplement: Supplementary file 7 — Source data Fig. 6 [file 44319_2025_625_MOESM7_ESM.zip › Figure 6/6B/6B'' (dCg-Gal4:+; +)-Thoracic_abdB.tif]

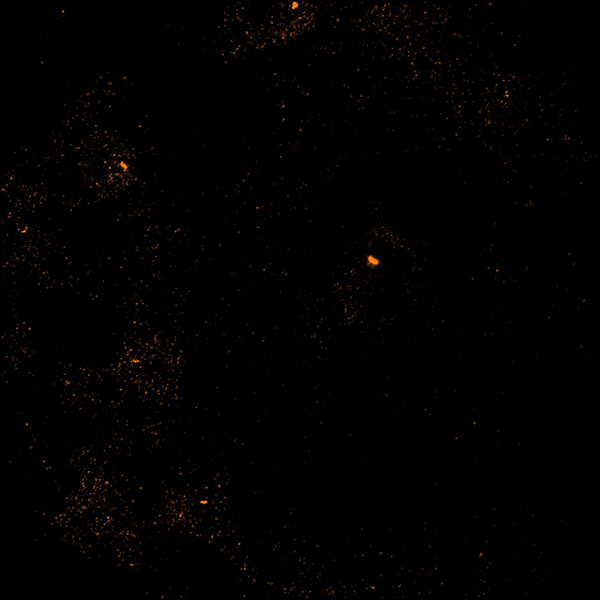

Supplement: Supplementary file 7 — Source data Fig. 6 [file 44319_2025_625_MOESM7_ESM.zip › Figure 6/6E/6E'' (dCg>AxnRNAi;+)-Abdominal_abdB.tif]

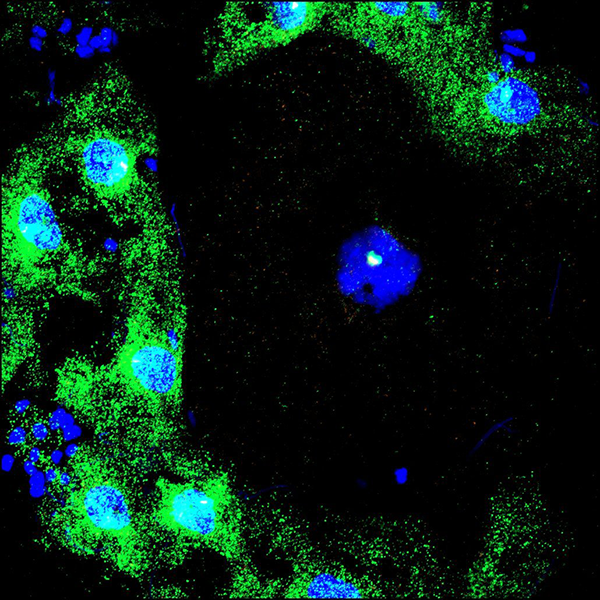

Supplement: Supplementary file 7 — Source data Fig. 6 [file 44319_2025_625_MOESM7_ESM.zip › Figure 6/6E/6E (dCg>AxnRNAi;+)-Abdominal_Composite.tif]

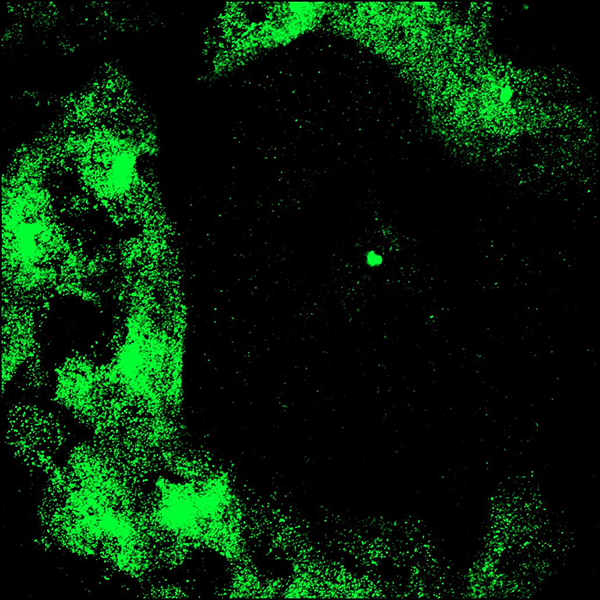

Supplement: Supplementary file 7 — Source data Fig. 6 [file 44319_2025_625_MOESM7_ESM.zip › Figure 6/6E/6E' (dCg>AxnRNAi;+)-Abdominal_abdA.tif]

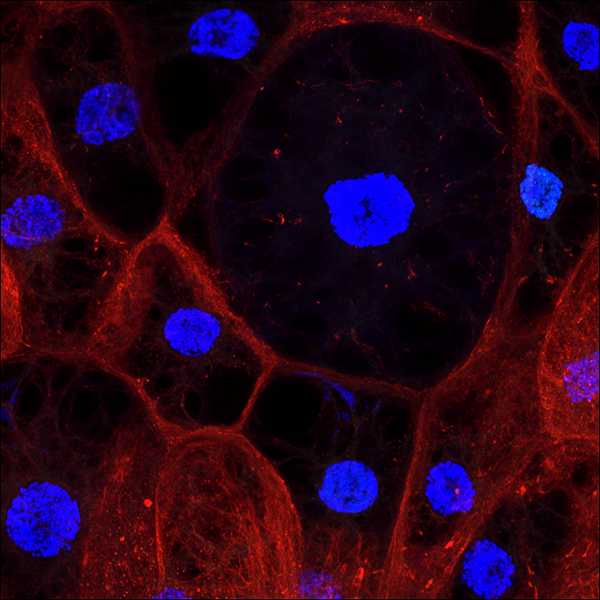

Supplement: Supplementary file 7 — Source data Fig. 6 [file 44319_2025_625_MOESM7_ESM.zip › Figure 6/6K/6K (dCg>AxnRNAi; Abd-B EGFP:+)-Abdominal.tif]

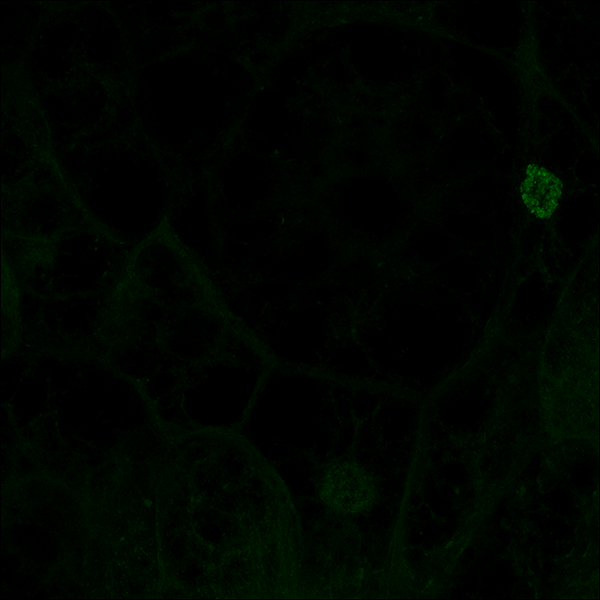

Supplement: Supplementary file 7 — Source data Fig. 6 [file 44319_2025_625_MOESM7_ESM.zip › Figure 6/6K/6K' (dCg>AxnRNAi; Abd-B EGFP:+)-Abdominal_Green Channel.tif]

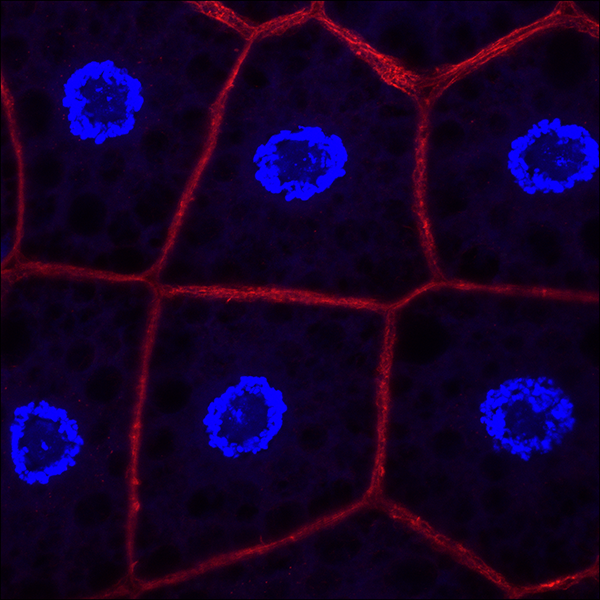

Supplement: Supplementary file 7 — Source data Fig. 6 [file 44319_2025_625_MOESM7_ESM.zip › Figure 6/6J/6J (dCg-Gal4:+; Abd-B EGFP:+)-Abdominal.tif]

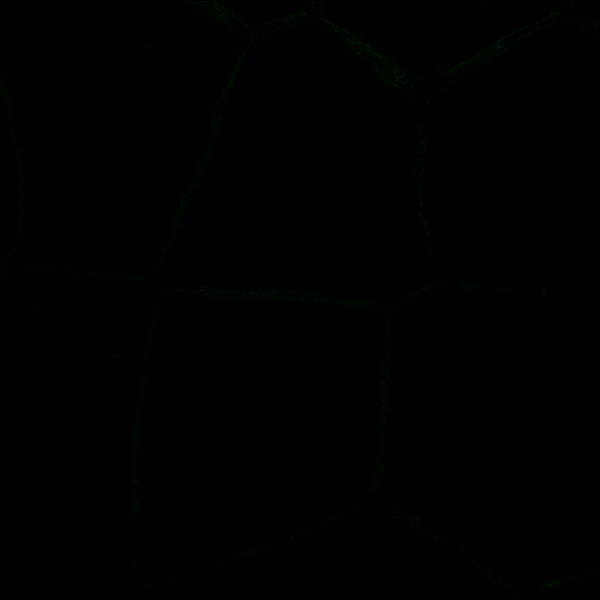

Supplement: Supplementary file 7 — Source data Fig. 6 [file 44319_2025_625_MOESM7_ESM.zip › Figure 6/6J/6J' (dCg-Gal4:+; Abd-B EGFP:+)-Abdominal_Green Channel.tif]

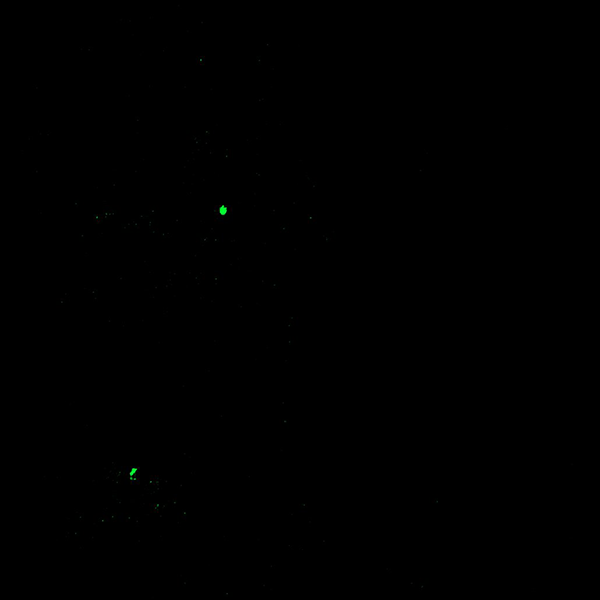

Supplement: Supplementary file 7 — Source data Fig. 6 [file 44319_2025_625_MOESM7_ESM.zip › Figure 6/6D/6D' (dCg>AxnRNAi;+)-Thoracic_abdA.tif]

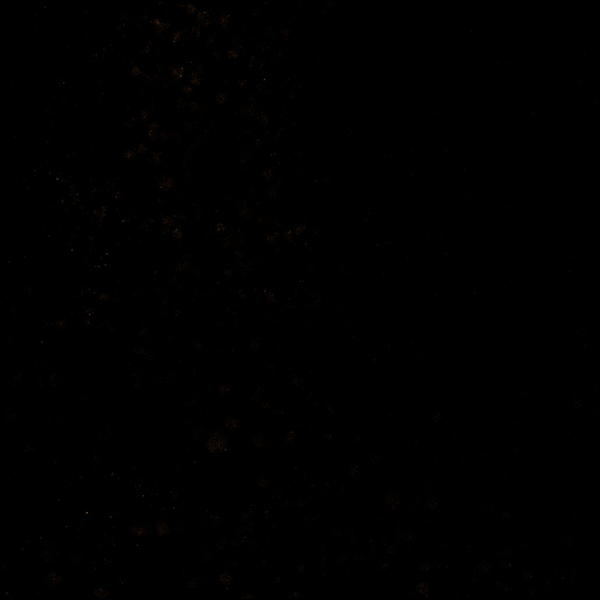

Supplement: Supplementary file 7 — Source data Fig. 6 [file 44319_2025_625_MOESM7_ESM.zip › Figure 6/6D/6D'' (dCg>AxnRNAi;+)-Thoracic_abdB.tif]

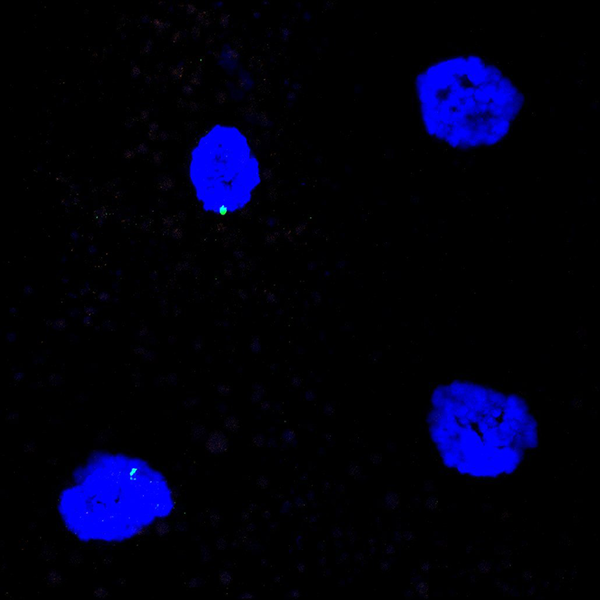

Supplement: Supplementary file 7 — Source data Fig. 6 [file 44319_2025_625_MOESM7_ESM.zip › Figure 6/6D/6D (dCg>AxnRNAi;+)-Thoracic_Composite.tif]

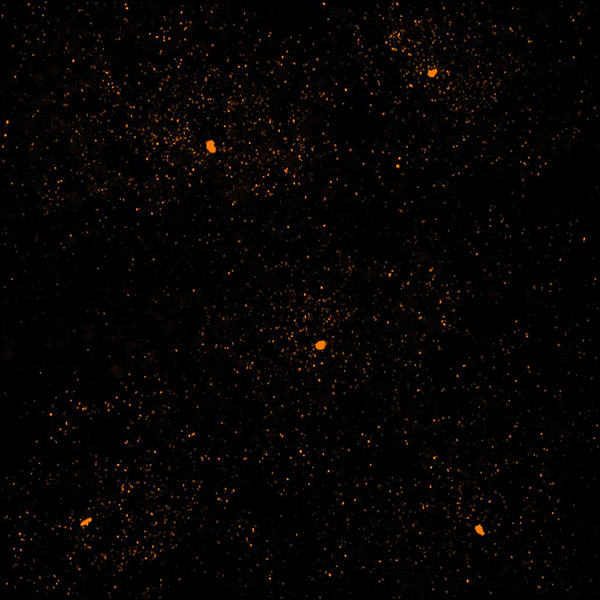

Supplement: Supplementary file 7 — Source data Fig. 6 [file 44319_2025_625_MOESM7_ESM.zip › Figure 6/6C/6C'' (dCg-Gal4:+; +)-Abdominal_abdB.tif]

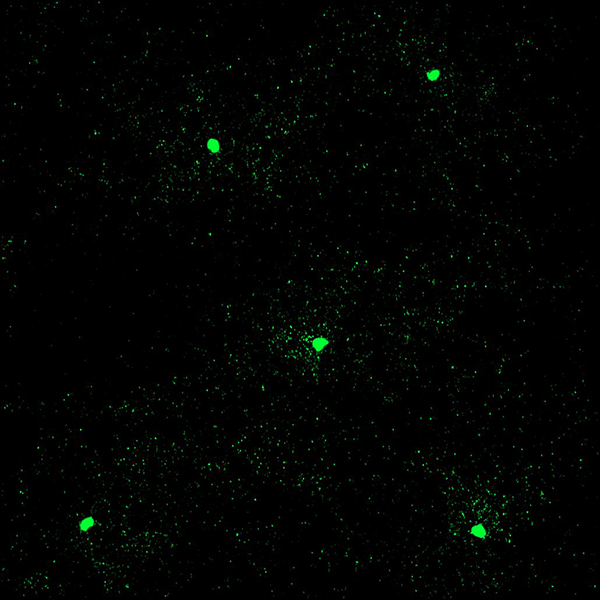

Supplement: Supplementary file 7 — Source data Fig. 6 [file 44319_2025_625_MOESM7_ESM.zip › Figure 6/6C/6C' (dCg-Gal4:+; +)-Abdominal_abdA.tif]

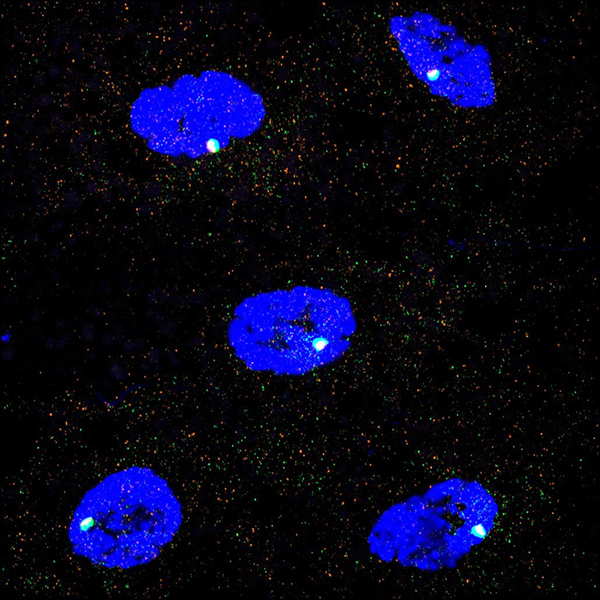

Supplement: Supplementary file 7 — Source data Fig. 6 [file 44319_2025_625_MOESM7_ESM.zip › Figure 6/6C/6C (dCg-Gal4:+; +)-Abdominal_Composite.tif]

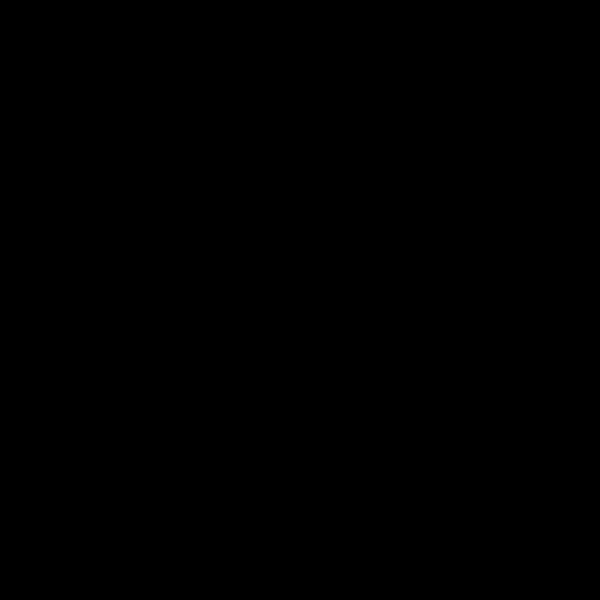

Supplement: Supplementary file 8 — Appendix Figure S5 Source Data [file 44319_2025_625_MOESM8_ESM.zip › Appendix Figure S5/S5F/S5F'_Green_Ubx EGFP (L3 wandering)_abdominal FB.tif]

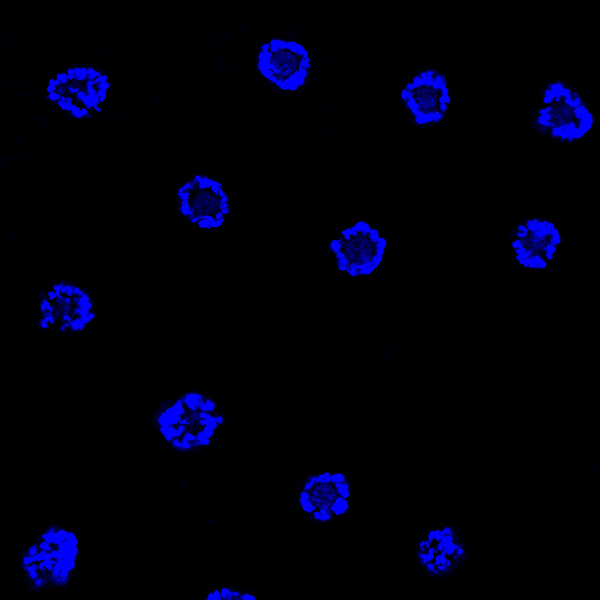

Supplement: Supplementary file 8 — Appendix Figure S5 Source Data [file 44319_2025_625_MOESM8_ESM.zip › Appendix Figure S5/S5F/S5F_Ubx EGFP (L3 wandering)_abdominal FB.tif]

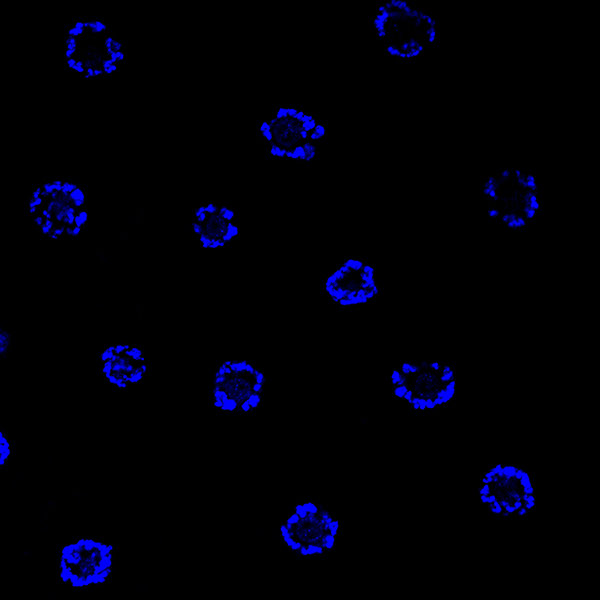

Supplement: Supplementary file 8 — Appendix Figure S5 Source Data [file 44319_2025_625_MOESM8_ESM.zip › Appendix Figure S5/S5E/S5E_Ubx EGFP (L3 wandering)_thoracic FB.tif]

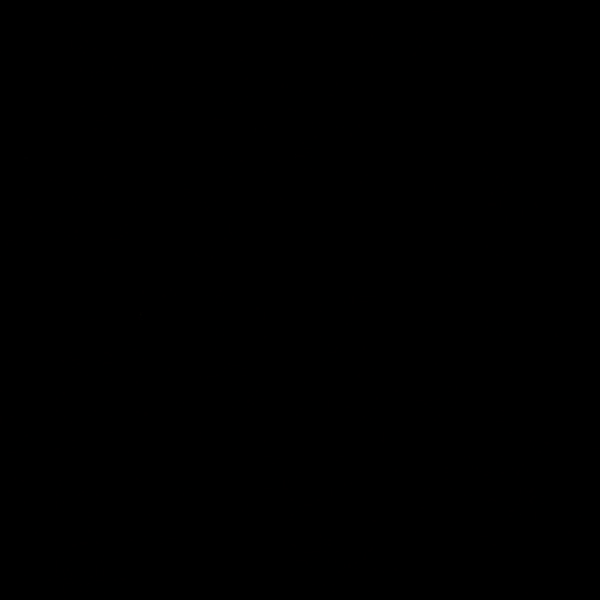

Supplement: Supplementary file 8 — Appendix Figure S5 Source Data [file 44319_2025_625_MOESM8_ESM.zip › Appendix Figure S5/S5E/S5E'_Green_Ubx EGFP (L3 wandering)_thoracic FB.tif]

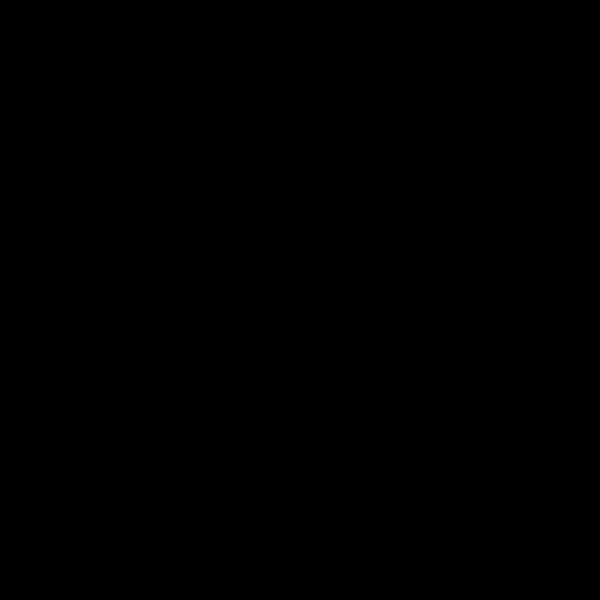

Supplement: Supplementary file 8 — Appendix Figure S5 Source Data [file 44319_2025_625_MOESM8_ESM.zip › Appendix Figure S5/S5B/S5B'_Green_Ubx EGFP (Early L3)_thoracic FB.tif]

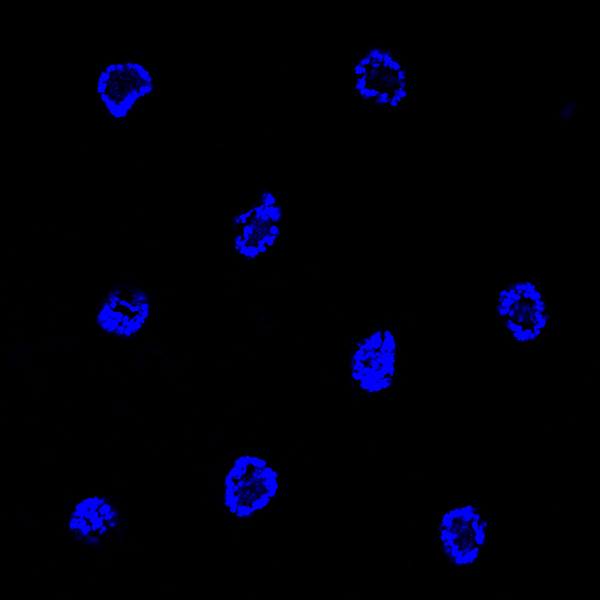

Supplement: Supplementary file 8 — Appendix Figure S5 Source Data [file 44319_2025_625_MOESM8_ESM.zip › Appendix Figure S5/S5B/S5B_Ubx EGFP (Early L3)_thoracic FB.tif]

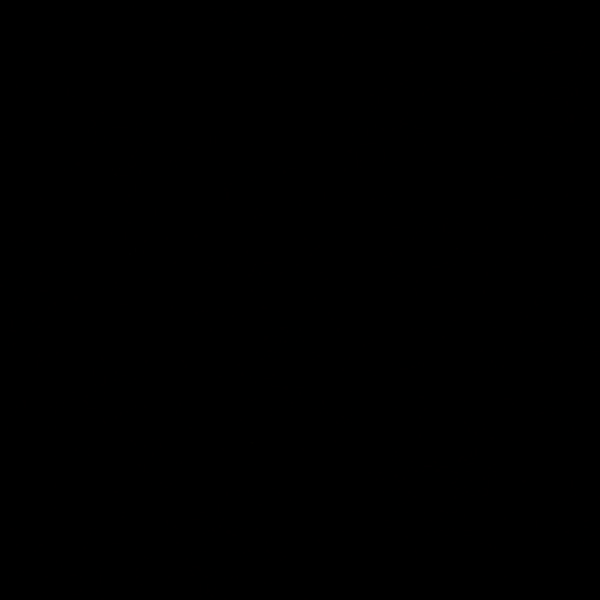

Supplement: Supplementary file 8 — Appendix Figure S5 Source Data [file 44319_2025_625_MOESM8_ESM.zip › Appendix Figure S5/S5C/S5C'_Green_Ubx EGFP (Early L3)_abdominal FB.tif]

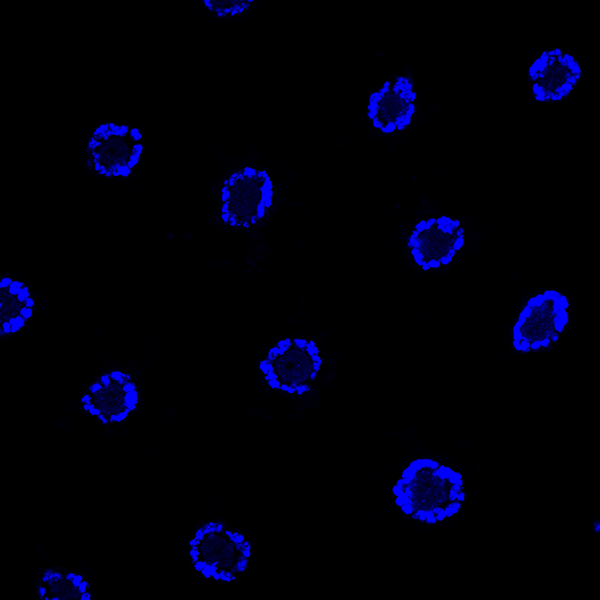

Supplement: Supplementary file 8 — Appendix Figure S5 Source Data [file 44319_2025_625_MOESM8_ESM.zip › Appendix Figure S5/S5C/S5C_Ubx EGFP (Early L3)_abdominal FB.tif]
